# Functional diversity of microbial ecologies estimated from ancient human coprolites and dental calculus

**DOI:** 10.1098/rstb.2019.0586

**Published:** 2020-10-05

**Authors:** David K. Jacobson, Tanvi P. Honap, Cara Monroe, Justin Lund, Brett A. Houk, Anna C. Novotny, Cynthia Robin, Elisabetta Marini, Cecil M. Lewis

**Affiliations:** 1 Laboratories of Molecular Anthropology and Microbiome Research (LMAMR), University of Oklahoma, Norman, OK, USA; 2 Department of Anthropology, University of Oklahoma, Norman, OK, USA; 3 Department of Sociology, Anthropology, and Social Work, Texas Tech University, Lubbock, TX, USA; 4 Department of Anthropology, Northwestern University, Evanston, IL, USA; 5 Department of Life and Environmental Sciences, University of Cagliari, Cagliari, Sardinia, Italy

**Keywords:** dental calculus, coprolites, microbiome, resilience, keystone, networks

## Abstract

Human microbiome studies are increasingly incorporating macroecological approaches, such as community assembly, network analysis and functional redundancy to more fully characterize the microbiome. Such analyses have not been applied to ancient human microbiomes, preventing insights into human microbiome evolution. We address this issue by analysing published ancient microbiome datasets: coprolites from Rio Zape (*n* = 7; 700 CE Mexico) and historic dental calculus (*n* = 44; 1770–1855 CE, UK), as well as two novel dental calculus datasets: Maya (*n* = 7; 170 BCE-885 CE, Belize) and Nuragic Sardinians (*n* = 11; 1400–850 BCE, Italy). Periodontitis-associated bacteria (*Treponema denticola*, *Fusobacterium nucleatum* and *Eubacterium saphenum*) were identified as keystone taxa in the dental calculus datasets. Coprolite keystone taxa included known short-chain fatty acid producers (*Eubacterium biforme, Phascolarctobacterium succinatutens*) and potentially disease-associated bacteria (*Escherichia*, *Brachyspira)*. Overlap in ecological profiles between ancient and modern microbiomes was indicated by similarity in functional response diversity profiles between contemporary hunter–gatherers and ancient coprolites, as well as parallels between ancient Maya, historic UK, and modern Spanish dental calculus; however, the ancient Nuragic dental calculus shows a distinct ecological structure. We detected key ecological signatures from ancient microbiome data, paving the way to expand understanding of human microbiome evolution.

This article is part of the theme issue ‘Insights into health and disease from ancient biomolecules’.

## 1. Introduction

Host-associated microbiomes are complex ecosystems with diverse sets of interactions between microbes, the host and abiotic features. Human microbiome research has primarily focused on documenting the genes/organisms present in a sample and differentiating microbiome communities using presence/absence and relative abundance data [1–5]. Such contributions have undoubtedly advanced the understanding of human biology; however, a stronger focus on taxonomic co-occurrence, identification of taxa with disproportionate influence on community function, as well as overall resilience of metabolic pathways will provide a more nuanced view of the microbiome. An analogy can be drawn from mammalian ecology in the United States’ Yellowstone National Park, where a focus on the role that wolves play as a keystone species yields greater clarification on cross-species interactions and dependencies. Wolves are not an abundant species in the ecosystem, yet their predator–prey relationships have tremendous downstream impacts on ecosystem production and stability [6–8]. A simple taxonomic inventory does not present the full picture of the wolves’ impact on the ecosystem, but an approach focused on their network of interactions demonstrates how they function as a keystone species that reshapes resource allocation and alters interspecies relationships throughout the ecosystem [6–8]. In the absence of deeper modelling, wolves would remain a rare biome variant, without a sophisticated understanding of their role as a keystone species.

Human microbiome research can clearly benefit from a similar approach but the momentum in applying ecological theory to the microbiome has only recently gained traction. In this vein, microbiome focus is slowly shifting from describing what is present in a microbiome to understanding the factors that drive community membership, polyspecies interactions, functional variation and ecosystem stability. While there is no unified or singular approach, ecological concepts such as community assembly, succession, response to disturbance, restoration, functional redundancy, response diversity, keystone taxa and genes, and co-occurring networks have made inroads in human microbiome research [9–19]. Each of these concepts leads to estimating ecological variables, providing a more nuanced view of human microbiomes, including how they are formed, what factors drive change, and which bacteria play important roles in maintaining homeostasis or stable conditions. It may appear that such a heavy focus on microbial ecology takes the focus away from human biology; however, there is growing support for the holobiont paradigm. Holobiontism posits that macro-organismal development, health, and general function relies on microorganisms, and therefore, microbes play a role in macro-organism ecology and evolution [20–23]; in other words, human-associated microbial ecology and human biology are inextricably intertwined.

The approaches for estimating and characterizing human microbiomes through ecological concepts are in an early stage of research but there have been numerous valuable insights. Community assembly- and succession-focused research has demonstrated that early life human gut microbiome composition is dynamic and strongly influenced by a variety of factors, including birth mode, nutrition and exposure to antibiotics [3,9,24,25], which can lead to downstream health effects [26]. Network analysis has been used to identify potential therapeutic avenues for *Clostridium difficile* infections in the gut [27] and evaluate gut microbiome structure and stability [19]. Taxonomic composition fluctuates over time in gut microbiomes [28,29] but evidence suggests there is stability in metabolic activity [30] that may be driven by functional redundancy [9,10,25]. Importantly, one should not consider resilience (and stability) as resilience to remain in a healthy state; ecosystems, and thus microbiomes, can be resilient to change when they are in an alternative state [31–34]. Similarly, keystone taxa need not be important for promoting a healthy state. For example, *Porphyromonas gingivalis* has been suggested as a keystone taxon for periodontitis because it alters immune system defence mechanisms and thus promotes a resilient periodontitis-inducing biofilm [35–37].

Stronger focus on ecological functions and interactions paints a more detailed picture of the role that taxa and functions play in different microbiome states. A logical next step is to apply these approaches to archaeological and palaeogenomic microbiome data. These data are in a most unique position to impact the ecological understanding of the human microbiome as they permit exploration of how human microbiomes have responded to major changes in the human condition, such as epidemiological transitions, colonialism, biogeographic range expansions, and industrialization [38–46]. In fact, the popularized roles the microbiome plays in human biology are deeply connected to ‘diseases of civilization', such as allergies, obesity, chronic inflammation, emerging infectious diseases, and the evolution of antibiotic resistance [1,47–51]. To understand the mechanisms behind these changes, we must know exactly what has changed in functional redundancy, keystone taxa, resilience and assembly of human microbiomes. While datasets from non-human primates and extant non-industrialized people provide some progress towards that goal, there is simply no line more intuitive to understanding ancestral microbiomes than to study ancient populations.

Ecologically focused microbiome research with ancient biomaterials (primarily coprolites and dental calculus) will present unique challenges, such as DNA degradation, small sample size, contamination and lack of time-series data. However, coprolites (i.e. desiccated faeces) and dental calculus (i.e. calcified dental plaque) have a long history of providing important information on human health and practices of the past and, in ideal conditions, preserve a record of the human microbiome [38,40,52–55]. The first ancient microbiome study to apply the next-generation DNA sequencing technology was largely centered around the premise of whether detailed taxonomic information from ancient human gut microbiome was retrievable, and if so, whether these resembled the contemporary human gut [56]. From an assemblage of pre-colonial coprolites from Mexico (Rio Zape), they observed a similar taxonomic profile to contemporary gastrointestinal (GI) tract microbiomes at the phylum level, as well concordance with contemporary non-industrialized populations at the genus level owing to presence of *Treponema* and *Prevotella*, both of which are nearly absent from gut microbiomes of industrialized populations [56]. A follow-up study [41] noted that the Rio Zape assemblage may be a rare find because coprolites from other archaeological sites, including coprolites directly extracted from well-preserved mummies, had very poor gut microbiome preservation, including a taxonomic profile that is not expected from any mammalian gut, let alone a human gut. Additionally, the Rio Zape coprolites are unique because of the relatively high number (*n* = 8) of samples with human GI microbiome signatures as compared to those from other archaeological sites [57].

Dental calculus has proved to be more reliable in reconstructing an accurate microbiome signature compared to coprolites [42,43,58] primarily because mineralization during life makes calculus a sturdy and rigid material lacking in organic nutrients [59,60]. Thus, dental calculus is more resistant to environmental contamination [59,60]. Often, more than 90% of the bacterial DNA found in dental calculus originates from known oral bacteria, whereas less than half of coprolite bacterial DNA originates from known gut microbes [41,58], and many of the challenges associated with studying coprolite microbiomes (including low DNA yields, soil contamination, and lack of a true human microbiome community) are less severe in ancient dental calculus. The first next-generation sequencing study of ancient dental calculus demonstrated that the oral microbiome could be reconstructed by amplifying the 16S rRNA gene from samples ranging from 5500 BCE–1600 CE [61]; however, the use of 16S rRNA variable regions has been shown to be problematic for ancient microbiome datasets owing to primer bias [62]. Shotgun metagenomic approaches face fewer biases for taxonomic identification and additionally allow for the reconstruction of genomes and functional characterization [45]. Along this line, metagenomics has been used to reconstruct genomes from *Tannerella forsythia* [45] and *Methanobrevibacter oralis* [46] as well as track diversity in functional and taxonomic profiles in the mammalian oral microbiome over time [43,61,63].

Here, we present an ecologically focused analysis on previously published Rio Zape human coprolites (*n* = 8, 700 CE) [41,57] and historical dental calculus samples from the Radcliffe Infirmary Burial Ground, UK (*n* = 44, 1770–1855 CE) [63], as well as novel metagenomic dental calculus data generated as part of this study (see the electronic supplementary material, Methods) from Maya individuals from Belize (*n* = 7, 170 BCE-885 CE) and Nuragic individuals from Sardinia, Italy (*n* = 11, 1400–850 BCE). To best adapt ecological approaches to ancient coprolites and dental calculus, we focused on analysing the structure and properties of microbiome networks, identification of keystone taxa and functional diversity of specific functions of interest. Each of these can be evaluated without time-series data. Compositionally, corrected networks using SparCC [64] were generated following the protocol suggested by Layeghifard *et al*. [65] using species-level bacterial taxonomic inventories from MetaPhlAn2 [66]. Each network was generated 100 times to estimate network properties (number of clusters, modularity, transitivity and articulation points). Modularity and transitivity values were categorized as very low, low, medium, high and very high based on the distribution modularity and transitivity values across the different networks we generated. Keystone taxa were identified using three techniques common to network evaluation: page rank [67,68], hubs [69,70] and closeness centrality [71,72]. Functional redundancy was evaluated with gene-level inventories generated by HUMAnN2 [73] using the UniRef [50,74] database. Finally, we compared the results from the ancient datasets to modern human microbiome datasets to evaluate our ability to take a deeper ecological approach with the former as well as to identify possible changes in microbiome structure and resilience between ancient and modern microbiomes. A more in-depth discussion of our methods and the techniques used for network analysis and keystone taxa identification can be found in the electronic supplementary material.

## 2. Results

### (a) Coprolites

#### (i) Network analysis

**Figure 1.**
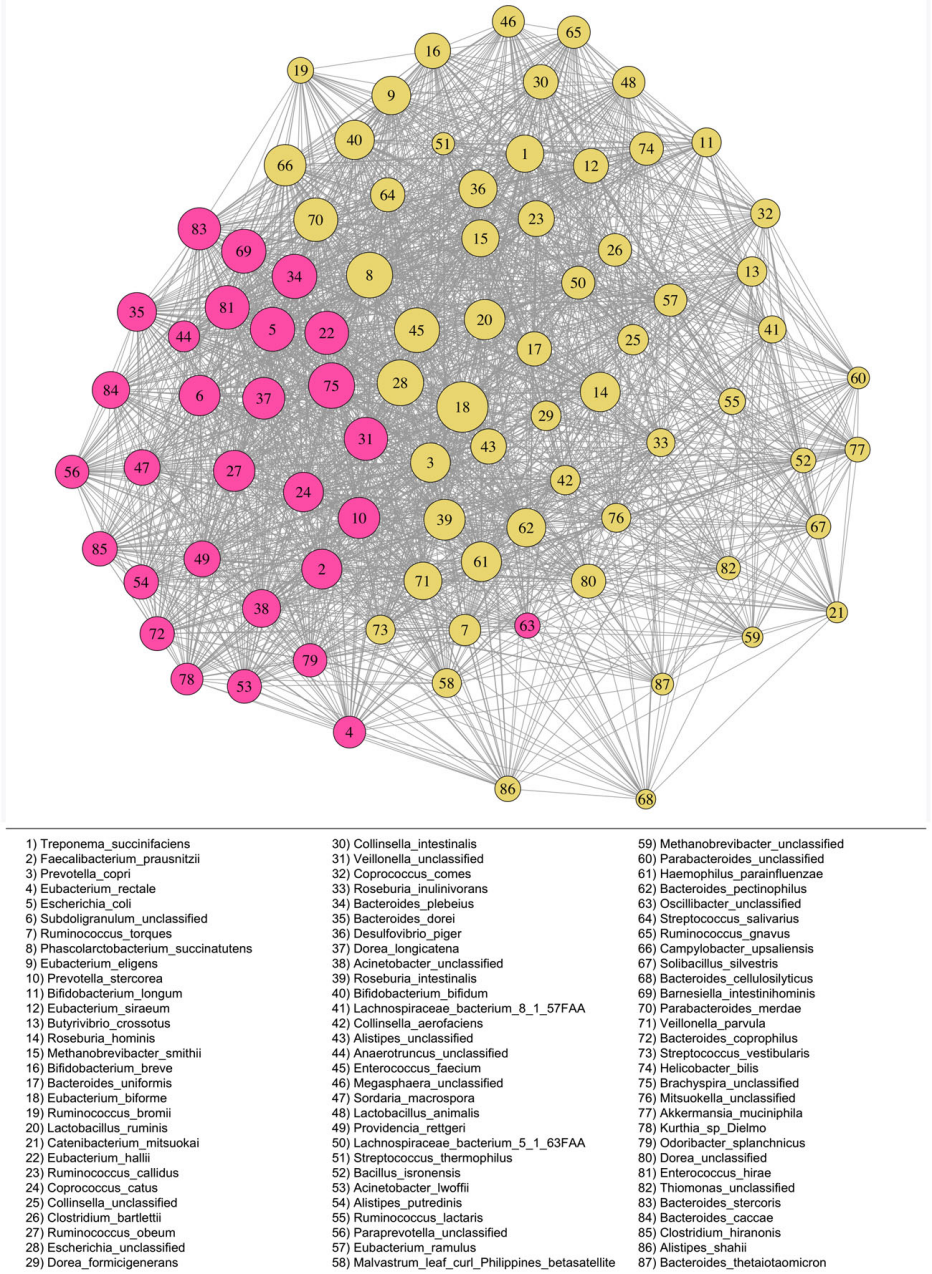
Rio Zape coprolite network (*n* = 8) generated with SparCC. Clusters are differentially coloured, keystones are outlined in black, and edges between nodes represent Pearson correlations greater than 0.3. Refer to legend for taxa corresponding to each numbered node. Clusters and nodes are highly interconnected, which is consistent with the low modularity and transitivity values observed.

We find a mean of 2.09 clusters (table 1) across the network for the Rio Zape coprolites (figure 1). Taking a broader view of network properties, the low modularity (mean = 0.11) in the Rio Zape network, indicates that the two clusters are highly interconnected. Similarly, the high transitivity (mean = 0.67) demonstrates that nodes are highly connected to each other outside of central nodes. *Eubacterium biforme* and *Phascolarctobacterium succinatutens* were identified as potential keystone species in each of the approaches used to discover keystones (table 2). Reads mapping to each keystone taxon were authenticated as ancient using MapDamage 2.0 [75,76] (electronic supplementary material, figure S1*a*–*d*). *Escherichia* and *Brachyspira* were also identified as keystone taxa; however, species-level resolution could not be obtained. Using MetaPhlAn2, we determined the presence of gut-associated members of these genera, such as *Escherichia coli* and *Brachyspira pilosicoli*, respectively, in addition to other unclassified species.

**Table 1.** Network properties of ancient microbiome ecology datasets. (Modularity was defined as: very low (<0.1), low (0.1–0.15), medium (0.15–0.2), high (0.2–0.3) and very high (>0.3). Similarly, transitivity was defined as: very low (<0.4), low (0.4–0.5), medium (0.5–0.6), high (0.6–0.7) and very high (>0.7). All ancient datasets have low or very low modularity and high or very high transitivity.)

population	sample type	number of clusters	modularity	transitivity
Rio Zape (*n* = 8)	coprolites	2.09 (s.d. = 0.43)	0.111 (s.d. = 0.010)	0.667 (s.d. = 0.003)
Maya (*n* = 7)	dental calculus	2.64 (s.d. = 0.67)	0.052 (s.d. = 0.008)	0.822 (s.d. = 0.004)
Nuragic (*n* = 11)	dental calculus	2.71 (s.d. = 0.87)	0.102 (s.d. = 0.013)	0.704 (s.d. = 0.003)
Radcliffe (*n* = 44)	dental calculus	14.14 (3.3)	0.063 (s.d. = 0.006)	0.738 (s.d. = 0.002)

**Table 2.** Likely keystone taxa were identified using three approaches (page rank, closeness centrality and hubscore). (Each network was generated 100 times and in each iteration the five most likely keystones from each approach were saved. The table below represents taxa that appear in at least 80 of the 100 iterations. There is strong agreement for each dataset's keystones, regardless of approach used.)

population		likely keystone taxa			
Rio Zape (*n* = 8)	page rank	*Eubacterium biforme*	*Phascolarctobacterium succinatutens*	*Escherichia* unclassified	*Brachyspira* unclassified
	closeness centrality	*Eubacterium biforme*	*Phascolarctobacterium succinatutens*	*Escherichia* unclassified	*Brachyspira* unclassified
	hubscore	*Eubacterium biforme*	*Phascolarctobacterium succinatutens*	*Escherichia* unclassified	*Brachyspira* unclassified
Maya (*n* = 7)	page rank	*Fusobacterium nucleatum*	*Treponema denticola*		
	closeness centrality	*Fusobacterium nucleatum*	*Treponema denticola*	*Cardiobacterium_valvarum*	
	hubscore	*Fusobacterium nucleatum*	*Treponema denticola*	*Cardiobacterium_valvarum*	
Nuragic (*n* = 11)	page rank	*Eubacterium saphenum*	*Olsenella* unclassified	*Streptococcus gordonii*	
	closeness centrality	*Eubacterium saphenum*	*Olsenella* unclassified		
	hubScore	*Eubacterium saphenum*	*Olsenella* unclassified		
Radcliffe (*n* = 44)	page rank	*Treponema socranskii*			
	closeness centrality	*Treponema socranskii*	*Tannerella forsythia*		
	hubScore	*Treponema socranskii*	*Tannerella forsythia*	*Neisseria elongata*	

#### (ii) Keystone functions

The functional roles of these keystones were interpreted by identifying the top 50 most abundant genes found in each taxon. Each keystone taxon has a high abundance of typical housekeeping genes, such as genes involved in synthesis of ribosomal RNA, transferases and transcriptional regulators (electronic supplementary material, table S1). We also identified genes involved in antibiotic-resistance mechanisms (MATE efflux proteins in *Brachyspira, E. biforme,* and *P. succinatutens*, and acriflavin-resistance proteins in *Brachyspira* and *P. succinatutens*). Toxin-antitoxin proteins were abundant in *Escherichia*, and transposases were abundant in all the keystone taxa (electronic supplementary material, table S1).

#### (iii) Functional redundancy

**Figure 2.**
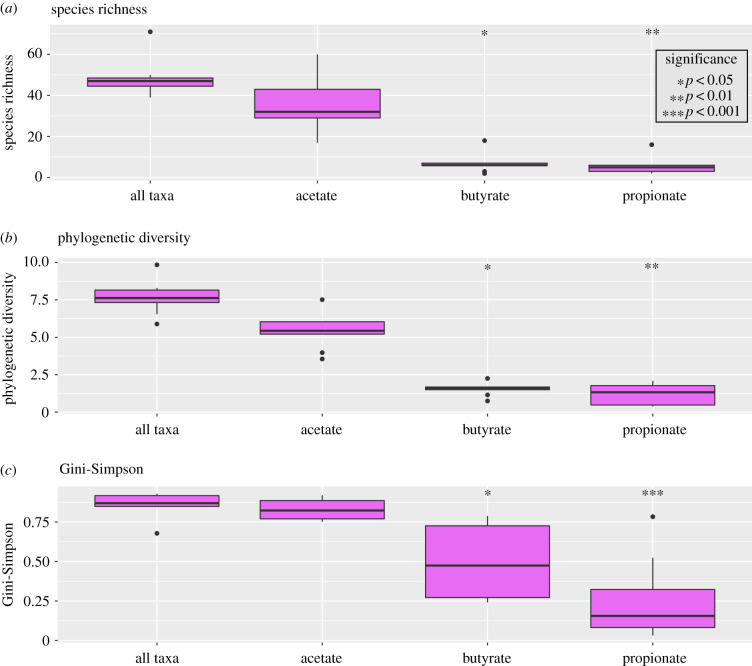
Functional diversity in the Rio Zape coprolites for short-chain fatty acid synthesis. (*a*) High functional redundancy (richness), (*b*) response diversity (phylogenetic diversity), and (*c*) evenness (Gini-Simpson) are observed for acetate, indicating production of acetate was more resilient than butyrate and propionate in the Rio Zape population. Taxa encoding butyrate are more evenly distributed than those encoding propionate.

We used a gene-centric approach to evaluate functional redundancy and response diversity as estimators of resilience in the coprolite microbiome. Short-chain fatty acids (SCFAs) such as acetate, butyrate and propionate, are critical for maintaining a properly functioning human gut microbiome [77–79] and therefore are an intuitive starting point for investigating gene-level functional diversity. We focused our analysis on three SCFA synthesis genes: acetate kinase (acetate), butyrate kinase (butyrate) and methylmalonyl-CoA decarboxylase (propionate). We observe higher diversity for acetate kinase in species richness (*p*-value <4 × 10^−7^), phylogenetic diversity (*p*-value <2 × 10^−9^) and Gini-Simpson (*p*-value <0.003) (figure 2*a–c*). These results point towards high response diversity (high number of phylogenetically diverse species) and more evenly distributed production for the taxa encoding acetate kinase, resulting in functionally redundant production of acetate kinase in the Rio Zape coprolites. Butyrate kinase and methylmalonyl-CoA decarboxylase are similar to each other in species richness and phylogenetic diversity (*p*-value >0.05), while Gini-Simpson is higher for taxa encoding butyrate kinase (*p*-value <0.03). Higher Gini-Simpson index values for butyrate production, compared to propionate, suggests a more even distribution of taxa encoding butyrate kinase and therefore greater protection against shifts in taxonomic abundance that may ultimately cause a decrease in propionate production.

### (b) Dental calculus

Shotgun metagenomic data were generated for Maya individuals (*n* = 7) and Nuragic individuals (*n* = 11). SourceTracker2 [80] analysis showed preservation of the oral microbiome signature in all samples, as evidenced by the proportion of reads attributed to taxa commonly found in subgingival or supragingival plaque (electronic supplementary material, figure S2).

#### (i) Network analysis

**Figure 3.**
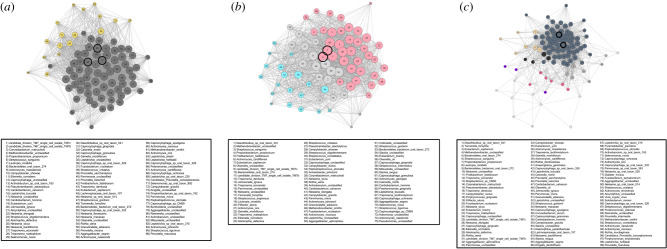
Networks for the three dental calculus datasets: (*a*) Maya, (*b*) Nuragic and (*c*) Radcliffe. Clusters are differentially coloured, keystones are outlined in black, and edges between nodes represent Pearson correlations greater than 0.3. Refer to legend for taxa corresponding to each numbered node. The high number of clusters in the Radcliffe network is probably related to increased sample size in this dataset. Highly interconnected clusters and nodes in each network is consistent with the low modularity and transitivity values observed.

The two archaeological populations show similar network properties: the Maya population (figure 3*a*) shows an average of 2.64 clusters, modularity of 0.052 and transitivity of 0.822. The Nuragic population (figure 3*b*) shows 2.71 clusters, modularity of 0.102 and transitivity of 0.704 (table 1). Modularity is significantly higher in the Nuragic population (*p*-value <2 × 10^−16^) and transitivity is higher in the Maya population (*p*-value <2 × 10^−16^), yet overall, both populations show very low or low modularity and very high transitivity compared to other networks generated in our analysis (table 3). Low modularity values are consistent with a network that has highly interconnected clusters; the clusters lack independence. Similarly, the high transitivity values reflect the diverse paths to connect the bacterial species in each network, providing further evidence of high interconnectivity in the network. The historical Radcliffe population (figure 3*c*) has significantly more clusters (14.1) compared to the Maya (2.64) and Nuragic (2.71) populations (*p*-value <2 × 10^−16^), which is probably driven by higher sample size in the Radcliffe dataset (see *Sample Size Simulation* section in Results). Despite this, the Radcliffe dataset is similar to the archaeological dental calculus in having very low modularity and very high transitivity (table 1).

**Table 3.** Basic network properties of the ancient and modern microbiome ecology datasets. (Modularity was defined as: very low (<0.1), low (0.1–0.15), medium (0.15–0.2), high (0.2–0.3) and very high (>0.3). Similarly, transitivity was defined as: very low (<0.4), low (0.4–0.5), medium (0.5–0.6), high (0.6–0.7) and very high (>0.7). Modern gut microbiomes datasets have higher modularity and low transitivity than the Rio Zape coprolites. Modern dental calculus is similar to ancient dental calculus. HMP, Human Microbiome Project.)

population	biological source	sample type	number of clusters	modularity	transitivity
Rio Zape (*n* = 8)	faeces	ancient coprolites	2.09 (s.d. = 0.43)	0.111 (s.d. = 0.010)	0.667 (s.d. = 0.003)
Matses (*n* = 26)		modern faeces	6.46 (s.d. = 1.57)	0.178 (s.d. = 0.017)	0.465 (s.d. = 0.004)
HMP, USA (*n* = 50)			17.03 (s.d. = 3.23)	0.379 (s.d. = 0.021)	0.268 (s.d. = 0.009)
Hadza (*n* = 25)			7.79 (s.d. = 1.65)	0.199 (s.d. = 0.014)	0.402 (s.d. = 0.009)
China (*n* = 38)			9.77 (s.d. = 3.29)	0.256 (s.d. = 0.015)	0.377 (s.d. = 0.005)
Maya (*n* = 7)	dental calculus	ancient dental calculus	2.64 (s.d. = 0.67)	0.052 (s.d. = 0.008)	0.822 (s.d. = 0.004)
Nuragic (*n* = 11)			2.71 (s.d. = 0.87)	0.102 (s.d. = 0.013)	0.704 (s.d. = 0.003)
Radcliffe (*n* = 44)			14.14 (3.3)	0.063 (s.d. = 0.006)	0.738 (s.d. = 0.002)
Spanish (*n* = 10)		modern dental calculus	2.67 (s.d. = 0.84)	0.101 (s.d. = 0.008)	0.632 (s.d. = 0.002)

The keystone species identified in each population are known oral taxa (table 2). Reads mapping to these taxa were authenticated as ancient for the Maya and Nuragic datasets on the basis of DNA damage patterns, generated using MapDamage 2.0 (electronic supplementary material, figures S3 and S4), suggesting that the networks represent an accurate ancient oral ecology. Additionally, keystone species were consistent regardless of analytical approach used (table 2). Taxa associated with periodontitis progression were identified as keystone in each of the populations: *Treponema socranskii* and *T. forsythia* in Radcliffe, *Eubacterium saphenum* and *Olsenella* sp*.* in Nuragic Sardinians, and *Fusobacterium nucleatum* and *Treponema denticola* in Maya*. Cardiobacterium valvarum* was also identified as a keystone in the Maya population.

#### (ii) Co-occurring taxa

We next evaluated the co-occurrence patterns of selected taxa of interest: early colonizing bacteria *Streptococcus gordonii*, *Streptococcus sanguinis* and *Actinomyces naeslundii* [13,81,82], as well as periodontitis-associated bacteria *T. forsythia*, *T. denticola* and *P. gingivalis* [13,83–86]*.* These bacteria were chosen to study cluster co-occurrence because they play an important role in ecological interactions and functions; early colonizers are among the first bacteria to colonize the dental surface and periodontitis-associated bacteria can shift the community to a disease state. We documented how often common members of the oral microbiome are found in the same cluster as each taxon of interest (electronic supplementary material, figure S5). In the Maya and Radcliffe populations, early colonizers like *S. gordonii* and *S. sanguinis* co-occur in the same cluster as the periodontitis-associated bacteria *T. forsythia* and *T. denticola*. In the Nuragic population, we observed a similar trend but *S. gordonii* does not co-occur with the other oral taxa and another early colonizer, *A. naeslundii*, appears to take *S. gordonii's* place. *Actinomyces naeslundii* does not co-occur with the above-mentioned oral taxa in the Maya and Radcliffe populations.

#### (iii) Keystone functions

Similar to the coprolites, the most abundant genes encoded by the keystones identified in each of the dental calculus samples include transporters, transferases and ribosomal proteins. Efflux-related proteins linked to antibiotic-resistance (MATE efflux and RND efflux) were identified as highly abundant genes in each of the Maya and Radcliffe keystones, but in neither of the Nuragic keystone taxa (electronic supplementary material, tables S2–S4). The Nuragic keystones both encode putative pathogenic genes: bacteriocin in *E. saphenum* and virulence activator in *Olsenella*. *Fusobacterium nucleatum* in the Maya samples was the only other keystone found to encode similar genes (haemolysin and ethanolamine utilization). Finally, the keystones *C. valvarum* (Maya), *T. denticola* (Maya) and *Olsenella* (Nuragic Sardinians) were found to encode toxin-antitoxin genes and other stress-response genes in high abundance.

#### (iv) Functional redundancy

**Figure 4.**
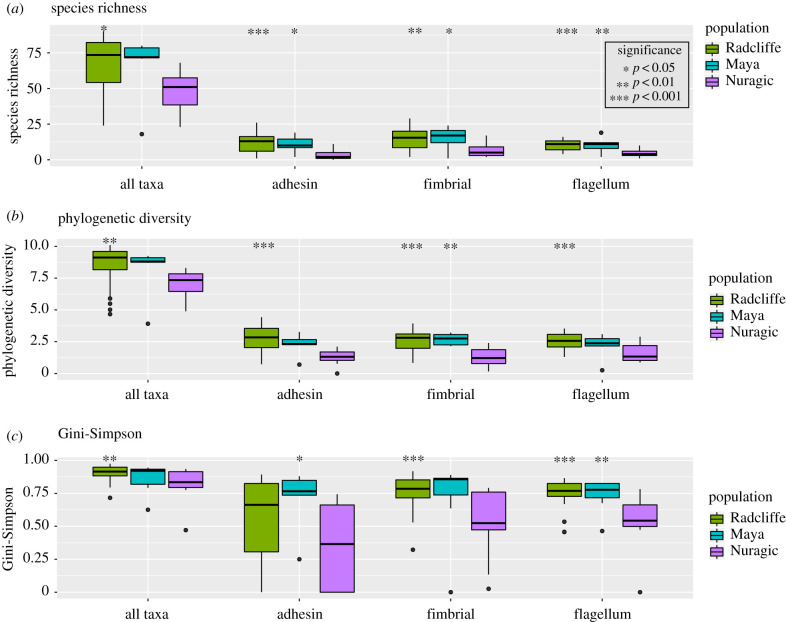
Functional diversity in the ancient calculus datasets for genes involved in bacterial cell adhesion and cell-cell binding. In general, the Maya and Radcliffe datasets have greater (*a*) functional redundancy, (*b*) response diversity and (*c*) evenness compared to the Nuragic samples for each gene of interest. These oral ecosystems may have been more robust in terms of dental calculus deposition and growth. Significant *p*-values are given in reference to the Nuragic dataset.

For gene-centric analyses, we focused on proteins involved in dental calculus formation via cell-cell binding (adhesins, flagellar and fimbrial proteins) [59] to give a better understanding of the functional redundancy of proteins involved in dental calculus formation. The Radcliffe and Maya populations have α diversity similar profiles for each binding protein and metric, while the Nuragic population has significantly lower richness than both Radcliffe and Maya populations for each gene (*p*-value <0.04; figure 4*a*). The Nuragic population has significantly lower phylogenetic diversity for every gene (*p*-value <0.0005) and Gini-Simpson for fimbrial and flagellum genes (*p*-value <0.0008) compared to Radcliffe (figure 4*b*,*c*). The Maya population has significantly greater phylogenetic diversity for fimbrial genes (*p*-value <0.003) and greater Gini-Simpson for adhesin and flagellum genes (*p*-value <0.03) as compared to the Nuragic Sardinians (figure 4*b*,*c*).

#### (v) Articulation points

Neither the coprolites nor dental calculus networks have articulation points. This is probably related to the low modularity in the networks: the interconnectivity of clusters within each network makes it likely for clusters to be connected to each other through multiple nodes.

### (c) Comparison to modern microbiomes

The coprolite and dental calculus networks inform about general properties (numbers of clusters, connectedness, and articulation points) and identify keystone species. We compared these data to modern microbiome datasets to assess the viability of ancient networks. The Rio Zape coprolites were compared to modern faecal microbiome datasets that represent hunter–gatherers (Hadza [87] and Matses [88]) and industrialized populations (MetaHIT-China [89] and Human Microbiome Project [90]). The ancient dental calculus was compared to modern Spanish dental calculus [63]. It is important to compare ancient dental calculus to modern dental calculus and not modern dental plaque, as dental calculus is distinct from dental plaque in maturation stage and ecology [63]. The small number of datasets mean that broad interpretations may be limited but it is a useful practice, nonetheless. The coprolite dataset showed fewer numbers of clusters (*p*-value <2 × 10^−16^), lower modularity (*p*-value <2 × 10^−16^), and higher transitivity (*p*-value <2 × 10^−16^) than modern faecal datasets (table 3). Unlike the low modularity and high transitivity found in the Rio Zape coprolites, the modern faecal microbiome networks had medium to very high modularity and low to very low transitivity (table 3). Both the modern and ancient dental calculus datasets each have low to very low modularity and high to very high transitivity, while a high number of clusters is only found in the Radcliffe ancient dental calculus dataset (table 3). The Human Microbiome Project faecal microbiome network was the only network that had articulation points. Keystone taxa were not shared between ancient and modern datasets, except for *P. succinatutens* serving as a keystone in the Rio Zape coprolites and modern Hadza hunter–gatherers.

The Rio Zape coprolites had similar response diversity and redundancy profiles when compared to the modern Matses and Hadza hunter–gatherers for taxa encoding SCFA synthesis genes. Overall, acetate kinase had the highest α diversity in each dataset, regardless of metric used (*p*-value <1 × 10^−6^) (electronic supplementary material, figure S6*a*–*c*). No significant differences were observed between the coprolites and Hadza (*p*-value >0.05), but the Matses hunter–gatherers had significantly greater phylogenetic diversity for propionate synthesis (*p*-value <4 × 10^−4^) and Gini-Simpson for butyrate synthesis (*p*-value <7 × 10^−10^) compared to the coprolites. The industrialized populations were significantly more diverse than the coprolites for all metrics in butyrate kinase and methmalonyl-CoA decarboxylase, as well as for species richness in acetate kinase (*p*-value <0.03). This observation is probably related to ascertainment bias that hinders annotation and taxonomic identification in non-industrial gut metagenomes [91], but this area bears further study.

The Spanish modern dental calculus had higher α diversity for all genes of interest in each metric when compared to the prehistoric Nuragic dental calculus (*p*-value <0.05) (electronic supplementary material, figure S6*d*–*f*). Likewise, the modern dental calculus had greater richness than the Radcliffe and Maya populations (*p*-value <0.012), with the exception of taxa encoding flagella in the Maya population. The modern dental calculus had significantly higher phylogenetic diversity than the Maya and Radcliffe datasets for fimbrial production (*p*-value <0.006) (electronic supplementary material, figure S6*e*) but there were no significant differences between the Radcliffe, Maya, and modern dental calculus in Gini-Simpson (electronic supplementary material, figure S6*f*).

#### (i) Sample size simulation

Most archaeological sites will provide small sample sizes. In our study, to address the effect of sample size on uncovering ecological interactions from human microbiomes, we simulated the effect of small sample size using modern GI tract microbiome data (see the electronic supplementary material, Methods). In brief, we randomly subsampled five, 10 and 20 samples from each dataset, then filtered taxa and generated the networks in the same way as we did for the full datasets. We found that both the number of clusters (*r*^2^ = 0.91) and network distinctness ratio (*r*^2^ = 0.88) increase with sample size (electronic supplementary material, table S5 and figure S7a*,b*). A high network distinctness ratio means high modularity and low transitivity, and therefore increased sample size leads to more clusters that are highly distinct from each other. We performed the same small sample size simulation with the Radcliffe ancient dental calculus dataset, which was our only dental calculus dataset with more than 20 samples. Similar to the faecal microbiome datasets, the number of clusters increased with sample size (*r*^2^ = 0.94); however, there was no increase in the network distinctness ratio (*r*^2^ = 0.52) (electronic supplementary material, figure S7*a*,*b*), indicating there are more clusters but those clusters are still highly interconnected. For both the faecal microbiomes and dental calculus, the keystones found in the full sample dataset were not found in any of the five-sample datasets and only rarely found in the 10-sample datasets (electronic supplementary material, table S6 and figure S8). The keystones identified in the 20-sample dataset were similar to the keystones found in the full datasets (electronic supplementary material, table S6 and figure S8).

The gene-specific approaches do not appear to be hindered by small sample size. As discussed above, we observed similar profiles between modern faecal datasets (Hadza, *n* = 26 and Matses, *n* = 25) and small coprolite datasets (Rio Zape = 8), as well as similar profiles between large ancient dental calculus datasets (Radcliffe, *n* = 44) and small ancient and modern dental calculus datasets (Maya, *n* = 7 and Spain, *n* = 10). Therefore, analysis of functional diversity in ancient human microbiome datasets remains robust even when few samples are recovered archaeologically.

## 3. Discussion

Archaeologists have made use of coprolites and dental calculus to study human biology, nutrition, and cultural behavior [38–40,42,43,58,92]. Applying ecological approaches to ancient human microbiomes from these materials is a clear next step to provide a deeper understanding of biology in the past. As research on modern human microbiome ecology is still in its infancy, it is expected that ancient microbiome ecology research will lag behind, but it should not be ignored. We have found that by focusing on ecological elements that can be interpreted from single time-point samples, such as ecological network properties, clusters of bacteria, keystone species, functional redundancy and response diversity, we can gain a glimpse of ecological interactions and functional diversity in ancient human microbiomes.

The four keystone taxa identified in the Rio Zape coprolites are known members of the contemporary human gut microbiome, which provides a validation for prehistoric keystone taxa. *Eubacterium biforme* and *P. succinatutens* are commensals that can produce the SCFAs butyrate [79,93] and propionate [79,94], respectively, in addition to performing other functions. The role of *Escherichia* in the gut is variable and has been identified in both disease and health-associated states [5,95]. Lastly, *Brachyspira* is primarily found in the GI-tract of pigs [96], chickens [97], and humans [98] and is associated with diarrhoea and other GI-tract maladies [99,100]; however, members of this genus can survive in soil for up to four months after faecal shedding [101]. While our analysis of keystone taxa was unable to provide species-level resolution for *Brachyspira,* a MetaPhlAn2 analysis showed that one of the species identified in the coprolites was *B. pilosicoli*. *Brachyspira pilosicoli* causes intestinal spirochaetosis in humans [98] and reads mapping to *B. pilosicoli* were authenticated as ancient using MapDamage 2.0, suggesting that *B. pilosicoli* could be a keystone species in this population. The diverse roles of the coprolite keystone taxa suggest that they may dominate distinct niches that lead to different impacts on human biology.

High response diversity and redundancy for acetate kinase is expected as acetate is the most abundant SCFA found in the human gut microbiome and is known to be encoded by diverse groups of bacteria [77–79,102]. Nevertheless, it is encouraging that we observed this trend in coprolites as further support that we picked up a gut microbiome profile. The lower Gini-Simpson values for methylmalonyl-coa decarboxylase indicates that a few species dominate production of propionate, while production of butyrate is more evenly distributed between taxa. From an ecological perspective, the Rio Zape ancient microbiomes were probably more prone to loss of propionate production than acetate and butyrate because only a few, non-phylogenetically diverse bacteria dominated propionate production.

The keystone taxa identified in the Radcliffe and Maya dental calculus datasets (*T. forsythia* and *T. socranskii* in Radcliffe and *T. denticola* and *F. nucleatum* in Maya) are members of the red and orange complex group of bacteria associated with periodontitis. Red complex bacteria are associated with driving periodontitis [86,103], and orange complex bacteria can function as bridging microbes that facilitate proliferation of red complex bacteria [104]. However, the simple presence of ‘orange' and ‘red' complex bacteria does not guarantee periodontitis progression, as the disease is complex [13,83,84,86]. Nevertheless, the presence of these bacteria as keystones, as well as other disease-associated keystone taxa in the Nuragic population (*E. saphenum* and *Olsenella* sp.) [83], indicates that such ancient oral microbiomes are prone to periodontitis. *Cardiobacterium valvarum,* found to be a keystone species in the Maya population, has been associated with endocarditis [105,106] and also has been isolated from the oral cavity of patients with periodontitis [106]. While we do not have any information on the cardiovascular health of the Maya individuals included in this study, the presence of *C. valvarum* in ancient dental calculus further supports the idea that the oral cavity has long hosted bacteria known to be involved in cardiovascular disease [45].

The Nuragic dental calculus is generally similar to the other two ancient dental calculus datasets for network properties; yet it is distinct in functional diversity and patterns of co-occurrence, highlighting the benefit of using multiple approaches to study ecological variation in the microbiome. There is significantly lower response diversity and redundancy in the Nuragic population for each gene of interest. These genes are involved in bacterial cell-cell binding and development of biofilms, which suggests that this population had unique ecological interactions during dental calculus deposition and growth. Along those lines, *S. gordonii* does not cluster with other oral bacteria and is replaced with *A. naeslundii* in the Nuragic Sardinian dental calculus. In the other datasets, *A. naeslundii* does not cluster with the other oral bacteria, while *S. gordonii* does. These two bacteria are early colonizers of the dental surface and therefore may represent alternative paths to early ecological interactions involved in ancient dental calculus formation.

The keystone taxa found in the coprolites and dental calculus are enriched for antibiotic resistant genes. The presence of antibiotic-resistance proteins in coprolites and dental calculus is anticipated; antibiotic-resistance is a natural result of millions of years of microbial evolution. However, it is noteworthy that three of the four keystone taxa in the Rio Zape coprolites are enriched for antibiotic-resistance proteins, suggesting how this mechanism may be important in a gut microbiome ecology. The Nuragic dental calculus was once again distinct owing to the lack of antibiotic resistant genes found in its keystone taxa, yet the Nuragic keystones were enriched for pathogenic genes. The explanation for why the Nuragic population exhibits a seemingly distinct oral ecological community remains elusive. Host genetics may play a role, as Nuragic Sardinians had very low genetic diversity [107] and host genetics does have an impact on the make-up of the human oral microbiome [108]; however, we did not analyse human genetics in our study and therefore we cannot provide further resolution for this idea. The unique oral microbiome in Nuragic Sardinians could also result from extensive use of copper mined from the island during the Bronze Age [109,110]. Copper has antimicrobial properties [111] and copper oxide, which is a product of heating copper [112] and has been found in Sardinian Bronze Age artefacts [113], is antimicrobial and has been shown to inhibit oral biofilm formation [114]. It is possible that copper affected Nuragic Sardinian oral microbiomes, such as through direct, accidental inhalation while working with the material or through copper leaching into water/food; however, we did not examine copper content of the dental calculus. These hypotheses may be of interest to future anthropological research.

The lack of articulation points in the ancient microbiome datasets means that there are no specific weak-link taxa that would result in a disconnected network if they were removed. Such flexibility in ecological structure can be beneficial but may also mean less stability in taxonomic and functional interactions. However, not much is understood about articulation points in microbiome networks, let alone ancient microbiome networks, and more work needs to be done to develop the theory in this area.

While this may point to greater ecological stability, it is more likely a result of flexibility in the network structure (meaning low modularity), which is directly tied to sample size.

Contemporary hunter–gatherers shared a keystone species with the coprolites (*P. succinatutens*). Additionally, the contemporary and ancient hunter–gatherer faecal microbiomes had similar response diversity and redundancy profiles for SCFA production. Both observations indicate a potential overlap in ecological community structure and function in contemporary and ancient hunter–gatherers. The similarity in ecological profiles is exciting because it also demonstrates that ecological interpretation is feasible with ancient microbiome datasets. A similar conclusion can be drawn from comparing the modern Spanish dental calculus to the Maya and Radcliffe ancient dental calculus. Each of these dental calculus datasets have low modularity, high transitivity, and similar phylogenetic diversity and Gini-Simpson values for each gene of interest. There are probably different factors driving similarity in ancient and modern gut microbiomes than the factors driving similarity in ancient and modern dental calculus, but these observations present opportunities for deeper investigations into how lifestyle changes over time influence variation in ecological interactions and functional redundancy within microbiomes.

While we were able to demonstrate key ecological signatures of ancient human microbiomes, there remains reason for caution in interpretation and application of ecological approaches to studying these biomaterials. A primary concern is sample size. In faecal datasets with small sample sizes (*n* < 10), we observed fewer clusters, lower modularity, and high transitivity. This pattern means that clusters will consist of many taxa and the clusters will be highly interconnected, which may obfuscate more nuanced ecological interactions. However, in the dental calculus dataset, we observed fewer clusters at small sample size, but no change in the network distinctness ratio with sample size, meaning increased sample size does not result in more separation between clusters and nodes. Nevertheless, both the faecal and dental calculus small-sample datasets report different keystone taxa than the respective full datasets. Given the current data available, archaeological studies with representative microbiome samples greater than 20 is strongly suggested for such analyses.

Unfortunately, excavating more than 20 coprolites with sufficient microbiome data to perform ecological analysis from a single site is unlikely. A major challenge is the presence of soil and non-GI tract bacteria in coprolites. Even in the best cases, human GI tract microbiome bacteria make up less than or equal to 75% of microbial DNA in coprolites [41]; thus, improved methods to isolate gut-derived molecules are required. Furthermore, among the coprolites that are consistent with the gut microbiome, the gut may not be solely human; for instance, dogs are coprophagic and suspected human coprolites may, in fact, be from dogs. Additionally, host DNA content may leach between coprolites, as well as other sources [115]. Fortunately, recently developed bioinformatic approaches are improving our ability to distinguish human from non-human coprolites [57,116]. Finally, coprolites are relatively delicate and often expose the sample to processes that alter DNA sequences and fragment nucleic acids. While microbiome data has been successfully recovered from coprolites in diverse sets of environments [41,117–119], we would expect such success to be an exception rather than the rule. Even when a set of coprolites do prove to retain a GI microbiome community, the small sample size may hinder ecological interpretation. Microbiomes from mummies initially provided an intuitive avenue to study ecology of ancient human gut microbiomes but was ultimately discovered to be misleading as the human gut, upon death, continues to be a moist, warm, enclosed bioreactor shaping the ecology to resemble that expected of compost [41]. Because ecology focuses so closely on taxa-taxa abundances and taxa-gene interactions, the preservation issues of a coprolites presents a major challenge, but Rio Zape proved to be an exception, as our results show that we can still study resilience and redundancy with small sample sizes.

A further challenge is authenticating that the communities are in fact ancient human microbiomes. Importantly, the previously published datasets used in this analysis validated their sequencing reads and we did the same for our newly generated data with SourceTracker2 [80], where the majority of our reads come from expected oral microbes. As expected for ancient DNA, all samples had reads which could not be assigned to known taxa in the database (categorized as ‘unknown' in SourceTracker2). We included these reads in our analyses, because a majority of them probably originate from ancient oral microbes but cannot be confidently assigned owing to existing databases being biased towards reference strains from modern, industrialized populations. These ‘unknown' reads may belong to taxa performing important functions and therefore removing them may bias results. An additional validation for the recovery of an ancient microbiome is to analyze post-mortem DNA damage patterns for reads mapping to the keystone taxa, by using programs such as MapDamage 2.0 [75]. Damage patterns consistent with ancient DNA lend strong credibility that the taxa at the centre of ecological interactions (i.e. the keystone taxa) are truly ancient, and not arising from recent contamination. Contamination from modern sources, either environmental or from human microbiomes during laboratory work, would be evident in both the types of microbes identified as keystone taxa, as well as a lack of the prototypical ancient DNA damage [120] in these keystones. Keystone taxa indicative of recent contamination would be bacteria found at high abundance in soil and/or human skin microbiomes. However, our results indicate that we are profiling an ancient microbial ecosystem because our keystone taxa are gut/oral microbes and have prototypical ancient DNA damage.

A greater interest in the maturing of ecological theory for microbiomes is needed, but applying such theory effectively requires a serious investment in mitigating ascertainment biases that burden current reference databases. Publicly available reference databases are skewed towards microbiomes from modern, industrial settings, of often health-associated microbiomes, which bias functional annotation of ancient and non-industrial studies. This ascertainment bias explains why we observe high taxonomic diversity for the industrialized gut microbiome datasets and provide at least a partial explanation of ‘unknown' reads in ancient dental calculus results. Our functional diversity approach relies on mapping to marker genes identified from reference taxa. Poor reference representation from non-industrialized populations will lead to bacterial genes and taxa being missed and categorized as ‘unknown'. Future microbiome initiatives must avoid exacerbating these biases, with an attention to data that informs, and contextualizes, the microbial ecology.

## Supplementary Material

Electronic Supplementary Material

## Data accessibility

Raw reads were deposited on NCBI SRA under the BioProject number: PRJNA657275.
