# Supplementary material for: Functional diversity of microbial ecologies estimated from ancient human coprolites and dental calculus
Source: Philos Trans R Soc Lond B Biol Sci. 2020 Oct 5;375(1812):20190586. doi: 10.1098/rstb.2019.0586 (PMC7702801; doi:10.1098/rstb.2019.0586)
Supplement: Electronic Supplementary Material [file rstb20190586supp1.docx]

**Electronic Supplementary Material**

This document includes:
I. Methods

II. Techniques for Network Analysis

III. Supplementary Figures

IV. Supplementary Tables

V. Supplementary References

**I. Methods**

*Archaeological Context of Novel Data*

Maya samples: The ancient Maya occupied northern Central America and parts of eastern Mexico from around 1000 BCE up contact with the Spanish in the 1500s CE, and their descendants still occupy the region today. The earliest Maya lived in small, widely scattered farming villages during the Preclassic period (1000 BCE-250 CE), and by the Classic period (250-830 CE) Maya villages, towns, and cities covered the region. During the Terminal Classic period (830-1000 CE), heartland of the Maya area experienced a significant disruption as the Maya political system collapsed and populations declined precipitously [1]. The seven Maya samples used in this study originate from burials at two sites in western Belize: Chan Chich, a moderately sized civic-ceremonial center, and Chan, a small farming community 50 km to the south. The Chan Chich samples derive from two burials (Burials CC-B12 and CC-B14) in the same building, Structure D-1, one of three structures in a small special-purpose courtyard near the site’s main plaza. Radiocarbon dating estimates Burial CC-B12 to cal. 713-885 CE, and Burial CC-B14 was interred slightly earlier based on burial context [2]. The Chan samples derive from Burials CH6 and CH19. Burial CH6 was recovered the principal building of the site ceremonial center (cal. 170 BCE-50 CE). The individual in Burial CH19 was interred in an L-shaped structure in the West Plaza of the site (cal. 570-660 CE) [3-5].

Nuragic Sardinia samples: The Nuragic period lasted from Middle Bronze Age to early Iron Age in Sardinia, Italy (~1600–800 BCE) and the Sardinian Nuragic population shows a typical early European farmer ancestry profile, although with a contribution from groups of the eastern Mediterranean and North Africa, related to the commercial trade networks existing with these populations [6]. The Nuragic society was substantially based on agriculture (cereals, legumes, grapes and figs) [7] and animal husbandry (sheeps, goats, cattle, pigs) [8], whereas evidence of aquatic foods is limited [9]. Collective burials were common as demonstrated by the minimum number of individuals found (MNI) at each of the archaeological sites from where the samples analyzed in this paper originated: Lu Maccioni (MNI = 40), Capo Pecora (MNI = 20), and Perdalba (MNI = 30) [10]. Lu Maccioni (Alghero) is a natural cave located at sea level in Northern Sardinia (cal. 1126-825 BCE). Capo Pecora (Arbus) is a natural cave located in Southern Sardinia, at 63 m above sea level (cal.1384-936 BCE), and Perdalba (Sardara) is located in Central Sardinia at 163 m above sea level. It is a collective burial structure of the so-called “domus de janas” (*home of the witches*), prehistoric artificial hypogea characteristic to Sardinia, and can be ascribed archaeologically to Nuragic times. All samples analyzed in this paper belong to the Nuragic osteological collection housed in the Sardinian Museum of Anthropology and Ethnography of the University of Cagliari [10].

*Shotgun-sequencing of ancient dental calculus samples*

Details of the dental calculus samples from Maya (n=7) and Nuragic individuals (n = 11) used in this study are provided in Supplementary Table 7. All samples were processed at the Laboratories of Molecular Anthropology and Microbiome Research (LMAMR) at the University of Oklahoma following established protocols for ancient DNA [11]. Up to 10 mg of dental calculus was used for DNA extraction, following an ancient DNA extraction protocol customized for dental calculus [12]. DNA libraries were built using established protocols [12] with a modification: DNA extracts were partially treated with uracil DNA-glycosylase (UDG) as given in [13]. Libraries were dual-indexed using the Kapa HiFi Uracil+ enzyme (Kapa Biosystems), quantified using the Fragment Analyzer (Agilent), and pooled in equimolar ratios. Size-selection was performed for a target range of 150-1000 bp using the PippinPrep (Sage Systems). Libraries were quantified using the Kapa Library Quantification kit (Kapa Biosystems) and sequenced on multiple runs (2 x 150 bp) of the Illumina HiSeq 3000 at the Oklahoma Medical Research Foundation, Oklahoma City, to an average of 16 million reads per sample (Supplementary Table 7).

*Data processing*

Previously published shotgun-sequencing data for ancient dental calculus samples from individuals from the historical Radcliffe Infirmary Burial Ground collection at Oxford, UK (n = 44) and ancient human coprolite samples from Rio Zape, Mexico (n=8) were downloaded from the European Nucleotide Archive (Supplementary Table 8). Previously published modern human microbiome datasets (fecal and dental calculus) were downloaded from NCBI. Participants with diabetes and/or inflammatory bowl disease were excluded from the MetaHIT-China dataset (n = 38) (Supplementary Table 8). Newly-generated as well as previously published data were processed using the same customized bioinformatics pipeline. Sequence reads were processed and merged using AdapterRemoval v2 [14], using a minimum overlap of 10. Reads were trimmed to remove Ns and low-quality bases and reads with a Phred score less than 30 were discarded.

*Assessing preservation of ancient microbiome signatures*

Post-processed reads were mapped to the GreenGenes [15] database of bacterial and archaeal 16S rRNA gene sequences using bowtie2 [16] with default parameters and the --no-unal option. Resulting SAM files were converted into BAM files, sorted, and duplicate reads were removed using SAMTools [17]. Custom scripts were used to generate a FASTA file comprising all the unique reads across all samples; this FASTA file was used as input for closed-reference OTU picking at a 97% identity threshold, implemented in QIIMEv1.9 [18] using uclust. Taxonomic inventories at the genus level were generated using QIIME scripts and were used as input for SourceTracker2 [19] to determine the proportion of reads attributed to oral, gut, and other sources.

*Authenticating ancient DNA*

Reads from keystone taxa identified for each population were authenticated as ancient using the program MapDamage 2.0 [20]. Post-processed reads for all samples in the population were separately mapped to the reference genomes of the keystone taxa identified for that population using bwa aln [21] with the following parameters: -n 0.03, -q 37, -l 1024, as suggested for ancient DNA [22]. Duplicate reads were removed using DeDup [23] and the resulting BAM files were used as input for MapDamage. When the keystone taxa was identified to the species level, that bacterial species was used as the reference genome for MapDamage. For the keystone taxa only identified to the genus level, we identified species belonging to the respective genus using MetaPhlAn and used this species as a reference for MapDamage: in the Rio Zape coprolites, we used *E. coli* for *Escherichia* and *B. pilosicoli* for *Brachyspira.* For the Nuragic dataset, we used *O. uli* for *Olsenella*

*Generating taxonomic and functional profiles*

Metagenomic taxonomic inventories were generated from the post-processed reads using default parameters in MetaPhlAn v2.0 [24]. Downstream analyses (networks, diversity) were conducted with the species-level data, excluding species with a mean abundance < 0.05% in each dataset. Functional profiles were generated from the post-processed reads using default parameters in HUMAnN2 [25] and the UniRef50 database [26]. The gene family output tables were used for downstream analysis after normalizing each sample’s gene abundance to copies per 1 million gene copies. These tables report abundance of each gene in every sample, as well as a stratified breakdown of how much each taxon contributes to the respective gene abundance.

*Network Analysis*

Filtered taxonomic tables for each sample in a dataset were combined into a single species-level taxonomic table for each dataset and were used as input for network generation in R [27]. SparCC networks were generated for each dataset following the protocol outlined by Laygerfield et al. [28] which accounts for compositionality in microbiome data by using sparse correlation coefficients. In brief, the input taxonomic table was used to create a sparse correlation coefficient matrix (using the SpiecEasi library) [29], which was in turn used as input to generate an undirected network implemented with the iGraph library [30]. Edges connecting nodes in our networks represent positive Pearson correlations >0.3. Networks were generated 100 times for each dataset in order to provide estimates of keystone taxa, modularity, transitivity, and number of clusters. Keystone species scores for the different approaches (HubScore, PageRank, and Closeness) were generated for each taxon in the resulting network with default settings from the iGraph library [30]. Keystone taxa with scores in the top 5 of all taxa were saved in each iteration of network generation, and the taxa that appeared in the top 5 in more than 80 of the 100 networks were determined to be potential keystone taxa. Cluster membership was determined using a walktrap algorithm, which performs random walks between nodes [30]. Modularity and transitivity for each network were determined with default parameters from iGraph [30] and range in value between 0 and 1. Categorical modularity groups were defined as: very low (< 0.1), low (0.1 – 0.15), medium (0.15 – 0.2), high (0.2 – 0.3) and very high (> 0.3). Categorical transitivity values were defined as: very low (< 0.4), low (0.4 – 0.5), medium (0.5 – 0.6), high (0.6 – 0.7) and very high (> 0.7). These values were determined by the distribution of modularity and transitivity values in each of the networks we generated and are meant to provide relative categories for these network attributes across the datasets we analyzed. We defined the network distinctness ratio as modularity divided by transitivity, as a way to measure how these variables change between datasets and sample size.

The number of clusters, modularity, and transitivity values reported in Tables 1 and 3 are mean averages from the 100 network iterations. The co-occurrence cluster (Supplementary Figure 5) represents the number of times (out of 100 network iterations) that common oral taxon along the y-axis is found in the same cluster as the taxon of interest across the x-axis based on cluster membership determined with the walktrap algorithm.

HUMAnN2 gene family tables were used to source gene abundance data from each keystone taxon. We calculated the average gene abundance in each sample for every UniRef50 annotation from each dataset in R. Within each dataset, the top 50 most abundant genes from the keystone taxa were used to evaluate potential functional importance of each keystone (Supplementary Tables 1-4).

*Functional Redundancy and Response Diversity Analysis*

Gene abundance for each gene, or gene group, of interest (acetate kinase, butyrate kinase, methylmalonyl-coa decarboxylase, fimbrial proteins, flagellar proteins, and adhesin proteins) were acquired from the HUMANn2 gene family tables for each dataset. Gene-abundance not attributed to any taxa (i.e. unclassified) was removed from downstream analysis because we were focused on the diversity of taxa encoding each gene. Gene-abundance per taxon tables were used to determine species richness, phylogenetic diversity, and Gini-Simpson. Species richness was calculated as the number of taxa encoding each gene. For phylogenetic diversity, we created a FASTA file comprising complete 16S rRNA gene sequences from the EzBioCloud database [31] for all the taxa identified in dental calculus and feces. These sequences were aligned using MAFFT with default parameters [32] and FastTree2 [33] was used within QIIMEv1.9 to create a phylogenetic tree, which was loaded into R. The vegan [34] and picante [35] libraries in R were used to calculate Gini-Simpson and phylogenetic diversity, respectively. The same process for calculating richness, phylogenetic diversity, and Gini-Simpson was used on the MetaPhlAn2 filtered output table to evaluate these metrics for the full community. Plots were generated in R using ggplot2 [36].

*Sample size analysis*

5, 10, and 20 samples were randomly subsampled in R from each of the modern fecal microbiome datasets. The MetaPhlAn2 taxonomic tables from each subsampling were filtered to remove taxa with < 0.05% mean abundance. Network generation and downstream analysis were performed in the same way as for the full dataset. The keystone taxa, number of clusters, modularity, and transitivity for each small sample size dataset was compared to the full datasets to evaluate the effect of small sample size on network properties.

*Statistical Test*

All tests for statistical significance were carried out in R. Where reported, p-values were determined with Kruskal-Wallis tests and false discovery rate correction [37].

**II. Techniques for Network Analysis**

There are various approaches that yield deep ecological understanding of microbiomes. In broad terms, time-series data are a strong resource for ecological approaches because they allow for tracking compositional changes and community stability for both genes and taxa. Assembly and succession can best be gauged by documenting taxonomic composition and functional changes over time, as distinguishing between transient and permanent microbiome residents requires a multi-sample approach [38-40]. Similarly, stability and resilience investigations benefit from an approach where samples are taken before and after some external challenge to the system, such as antibiotics. Ideally, this longitudinal data can be used to inform and predict about stability and resilience when only single time-point data are available. However, time-series data are logistically and monetarily challenging, and in the sense of a longitudinal clinical-like study, would be unheard of for archaeological studies. Nevertheless, ecological research into ancient microbiomes is still very much attainable with non-time-series data through the help of network analysis.

Co-occurring bacteria, keystone taxa, and community structure can be represented as an ecological network. Network analysis takes advantage of elements of graph theory to uncover relationships between members of complex communities, whether it be infrastructure networks, social networks, or biological ecosystems [41, 42]. Generating microbiome networks requires special attention due to the nature of sequencing data. Microbiome data are compositional due to an arbitrary maximum number of sequencing reads that can be generated on a NGS sequencing instrument [43] and this compositionality can lead to spurious relationships if it is not taken into account [43, 44]. Various methods have been developed to address compositionality in microbiome networks [29, 45-47] but in general, they use log-ratio transformations prior to downstream analysis [29, 43, 44, 46, 47]. In microbiome network analysis, each bacterial taxon forms an individual circular node and straight-line edges connect nodes, which represents correlations between two taxa and various cutoffs can be used to depict strength of correlations. In Supplementary Figure 9A, we present an example of a network with edges representing positive correlations >0.3. A node’s degree is the number of edges connected to that node and clusters are groups of nodes that share a high number of connections within the cluster and fewer connections outside the cluster. Identifying **nodes, edges, and clusters** are key tenets of network analysis [28, 38, 41, 48]. Network theory has applications in many fields of study, and is a broad and growing field, so here we focus on only a few important aspects that are easily translated to the characterization of a microbial ecology.

**Co-occurring clusters** of bacteria not only inform about which bacteria are co-dependent and interact with each other, but also inform about the structure of the microbial community. Knowing the structure of the microbiome is important ecologically because communities are made up of more than just singular interactions - there are niches and subgroups within larger communities. The taxonomic and functional nature of clusters can highlight how taxa are partitioned within the microbiome and potentially suggest spatial or niche segregation. Various methods have been created to detect clusters within a network but in general they work by simulating network clusters and choosing a network topology that optimizes modularity (strength of division into clusters) and transitivity (a measure of connectivity) of the network. Clusters are typically connected to other clusters in the network but can be isolated from the rest of the network**. Isolated clusters** may indicate a highly specialized set of taxa or functions.

**Modularity** [49, 50] is an important feature in uncovering microbiome structure as high modularity indicates there are many interactions within any given cluster, but few interactions between clusters, while low modularity indicates that there are many connections between clusters (Supplementary Figure 9B). In microbiomes with highly segregated functional groups and niches, we would expect high modularity as taxa within any given niche have few interactions with bacteria outside the niche. Similarly, we can interpret structure from the number and size of clusters within a microbiome. If there are few clusters with many bacteria, the community may have less specialized structuring and thus more fluidity in ecological function. **Transitivity** provides an indication of whether nodes already connected through a central node are likely to be connected to each other independent of that central node [28, 41]. High transitivity means many connections between bacteria and many routes to connect bacteria, suggesting a microbiome community with many different layers of interactions (Supplementary Figure 9C). Modularity and transitivity typically have an inverse relationship to each other, as networks with low modularity and high transitivity both signal high numbers connections between nodes.

Modularity, transitivity, and co-occurring clusters present a holistic view of the network structure; in other words, they are focused on emergent network properties. There is additional benefit to focusing on individual nodes in the network. A primary interest for microbiome ecology research is how individual taxa are connected to the rest of the network: are they at the center of the whole network, do they connect different clusters together, or are they isolated from taxa across the entire network? There are a multitude of approaches for identifying taxa central to the network and the most straightforward mechanism is by looking at its **degree**. Degree is simply a measure of how many connections a given node (bacterial taxon) maintains and it provides a quick way to identify highly and sparsely connected bacteria in the network. In our analysis, **Hub Score** can be thought of as an analogue to degree [41]. A slightly more nuanced approach is to use the **PageRank** algorithm, in which each node is given a weight depending on the quantity and quality of connections for each node but ultimately provides a similar interpretation as using degrees [41, 51, 52]. **Centrality** is a further method for determining how an individual node interacts with the remainder of the network [53]. **Closeness Centrality** depends on the use of paths, which trace the number of edges needed to connect any two nodes. High closeness nodes are those nodes that have the shortest average path length between itself and all other nodes in the network, meaning that it is central to the full network.

The connectivity of individual nodes in the network uncovers keystone taxa and taxa important for ecological stability. **Keystone taxa** have large influences on the microbiome community, independent of their relative abundance [54]. These bacteria may produce specific nutrients that are metabolized by other microbes or provide protection against environmental stressors. Keystone taxa are at the center of a network because they are not just important for one group of bacteria, but rather are important for the entire community to function. In microbiome networks, keystone taxa should be thought about as hubs for the network and thus are central to the full network. Therefore, we can determine keystone taxa using Hub Score, PageRank, and Closeness Centrality. There is a possibility that keystone identified by Hub Score and PageRank will differ than those identified through Closeness Centrality because the former two methods rely on number and quality of connections to each node, while the latter relies on tracing paths throughout the network. However, in most cases the keystone taxa identified by HubScore and PageRank are the same as those identified by using Closeness Centrality.

Outside of keystone taxa, other taxa can also have a large impact on the network by serving as **articulation points** [42]. Articulation points are nodes that connect two different clusters together and they are the only node that connect those two clusters. Articulation points are important for maintaining integrity in the network. Such taxa are often different from keystone species because often articulation points will have a small degree and be found more towards the periphery of the network; however, it is possible for an articulation point to have high centrality and serve as a keystone. Peripheral articulation points can be just as important as keystone taxa because removal of an articulation point can lead to disconnected clusters in the network [42]. Disconnected clusters may lead to instability and potentially a loss of resilience in the community.

Theory to Data Analysis – Functional Characterization

Networks are a path to reveal keystone taxa in microbiome, but they do not provide the detail of what, exactly, those keystone taxa do in the ecosystem. Some of the functional information may be gleaned from classic microbiology, where individual organisms were isolated and functional characterized, but for diverse ecological niches such as the gut and oral cavity, the majority of taxa have never been isolated in culture in part, because there are so many species to characterize, but also, in large part, because we know so little about them that attempts to find the substrates and conditions to grow them in isolation fail. The opportunity to find microbiome functional potential in ancient biomolecules has begun to benefit from work above the genome, such as information gleaned by the proteome [55] and metabolome [56] but there remains ample opportunity to characterize function via ancient DNA, from the metagenome.

Much like taxonomic inventories, metagenome data provide the opportunity for gene (or functional) inventories. Yet these inventories still provide little detail on interactions throughout the ecosystem. Using the Yellowstone analogy, presenting an inventory of the totality of functions performed in the ecosystem is a useful first step, but provides little detail on functional/spatial variation, resilience of behaviors, and interactions between taxa. Closer examination of which taxa perform specific functions, particularly the functions carried out by keystones, presents the opportunity to determine why certain taxa are keystones and detail how keystones influences an ecosystem. Additionally, this practice also facilitates an understanding of the potential for keystones to be replaced by other members of the same ecology that can assume those roles under disturbance. Bacterial keystone taxa may encode a wide diversity of genes and be thought of as “generalists'' or have a limited genome with more “specific” functional potential. A deeper understanding of keystone taxa and their functional capabilities presents an exciting prospect for understanding the ancestral states of human microbiomes, where the dynamics of keystone species and functional roles can be viewed in terms of major shifts in the human condition. Similarly, clusters of bacteria that are discovered via network analysis can be analyzed through their functional capabilities. Well-defined clusters may serve as distinct functional groups that specialize in production, or utilization, of specific metabolites and thus provide clearer evidence of ecological roles in the microbiome community.

From a gene-centric approach, alpha diversity (species richness, Gini-Simpson, Phylogenetic Diversity) of the taxa that perform specific gene functions give insight into functional redundancy and response diversity. When estimating the diversity of taxa associated with a specific gene, richness and Gini-Simpson estimate functional redundancy, while phylogenetic diversity estimates response diversity. Typically, these two estimates would be expected to be consistent, but exceptions are theoretically possible; for example, if there were a high number of taxonomically similar taxa encoding a single gene, then this gene would have high redundancy (via richness), because many taxa encode it, but low response diversity (via phylogenetic diversity) because the taxa are closely related to each other. Gini-Simpson serves as a marker of the relative abundance distribution of taxa performing a function (evenness), if there are many taxa but a few taxa are the dominant producers, then Gini-Simpson will be low (close to 0), while lack of dominant taxa leads to high Gini-Simpson (close to 1). High Gini-Simpson means high redundancy because the community is not at risk of losing one of the dominant producers of said function.

**III. Supplementary Figures**

Supplementary Figure 1A-D: Damage plots generated using MapDamage for the keystones identified in the Rio Zape coprolites. Red indicates C to T transitions and blue indicates G to A transitions. The Y axis shows the proportion of sites containing the nucleotide change and the X axis shows the position along the DNA fragment. *Escherichia* and *Brachyspira* keystones were not identified at the species level; however, *E. coli* and *B. pilosicoli* were both identified in the coprolite samples and were thus used as references in MapDamage*.* Damage patterns are consistent with ancient DNA for the Rio Zape coprolite keystones.


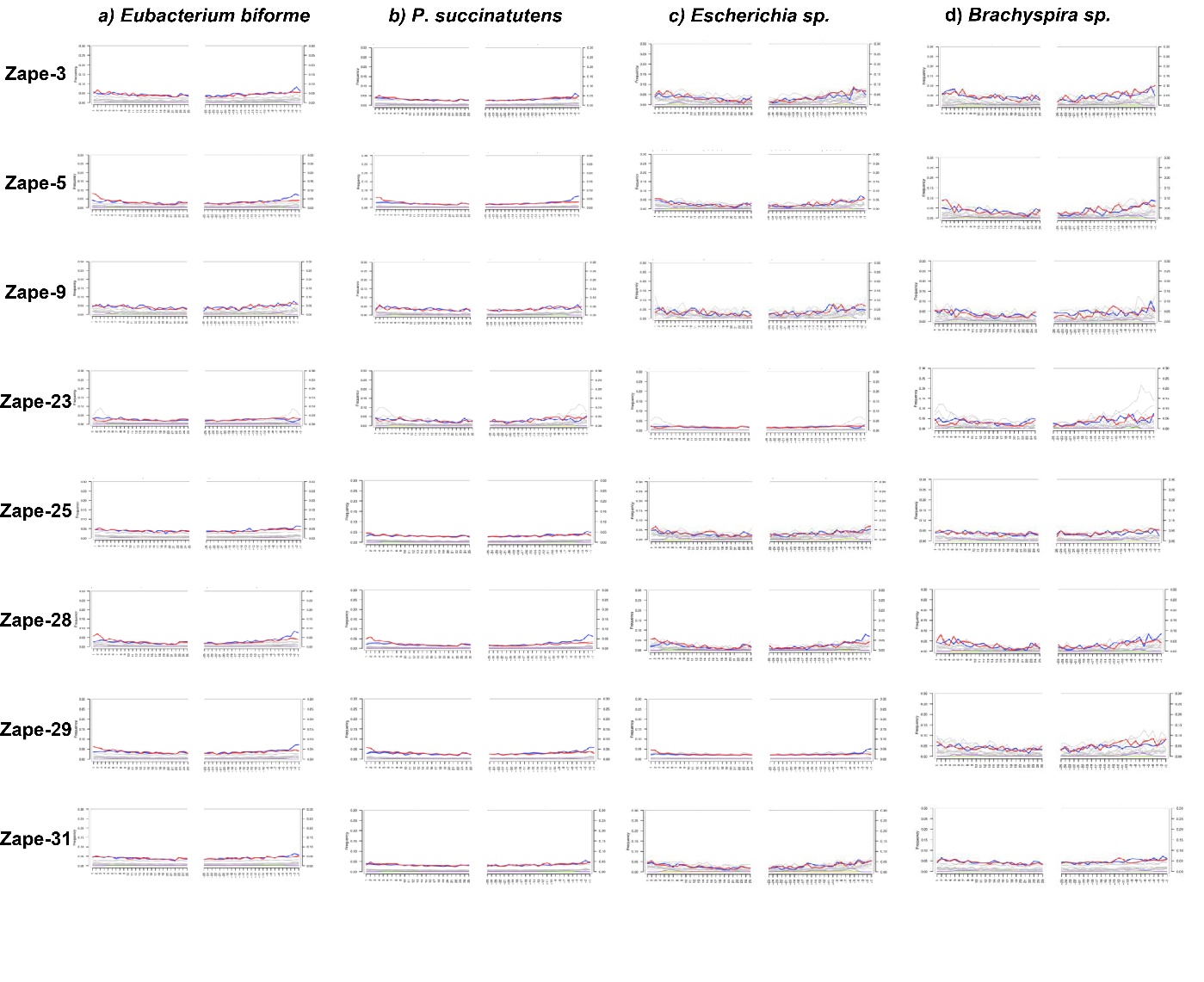


Supplementary Figure 2: Stacked bar plots of Bayesian SourceTracker results for the dental calculus samples from the Nuragic and Maya individuals. The Y axis shows estimated proportions of source contribution at the genus level, using modern subgingival and supragingival plaque, urban and rural gut, skin, and soil datasets as model sources.


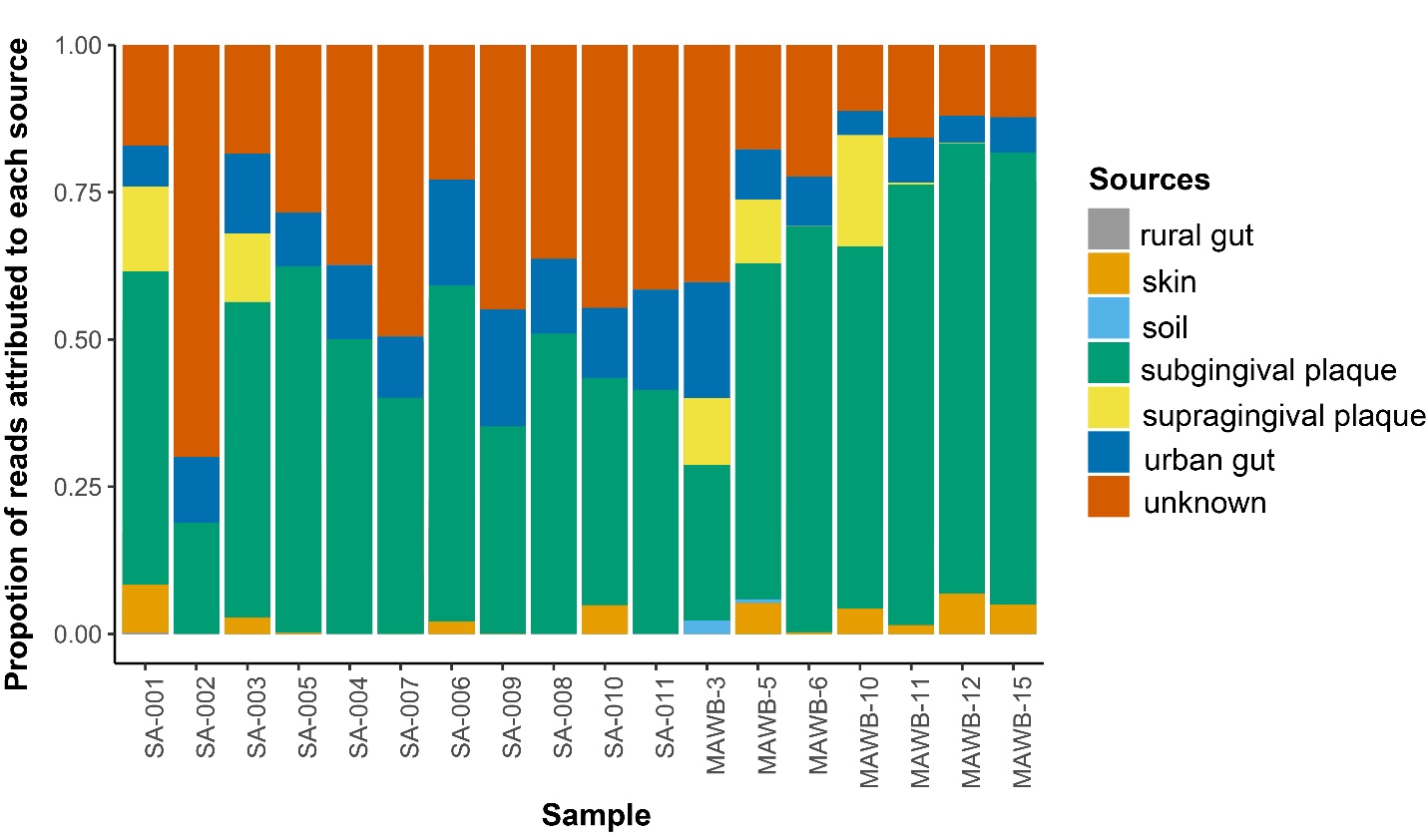


Supplementary Figure 3A-B: Damage plots generated using MapDamage for the keystones identified in Nuragic dental calculus. *Olsenella* was not identified at the species level as a keystone; however, *O. uli* was identified in the Nuragic samples and was thus used as the reference for MapDamage*.* A) *E. saphenum* and B) *O. uli* show damage patterns that are consistent with ancient DNA.


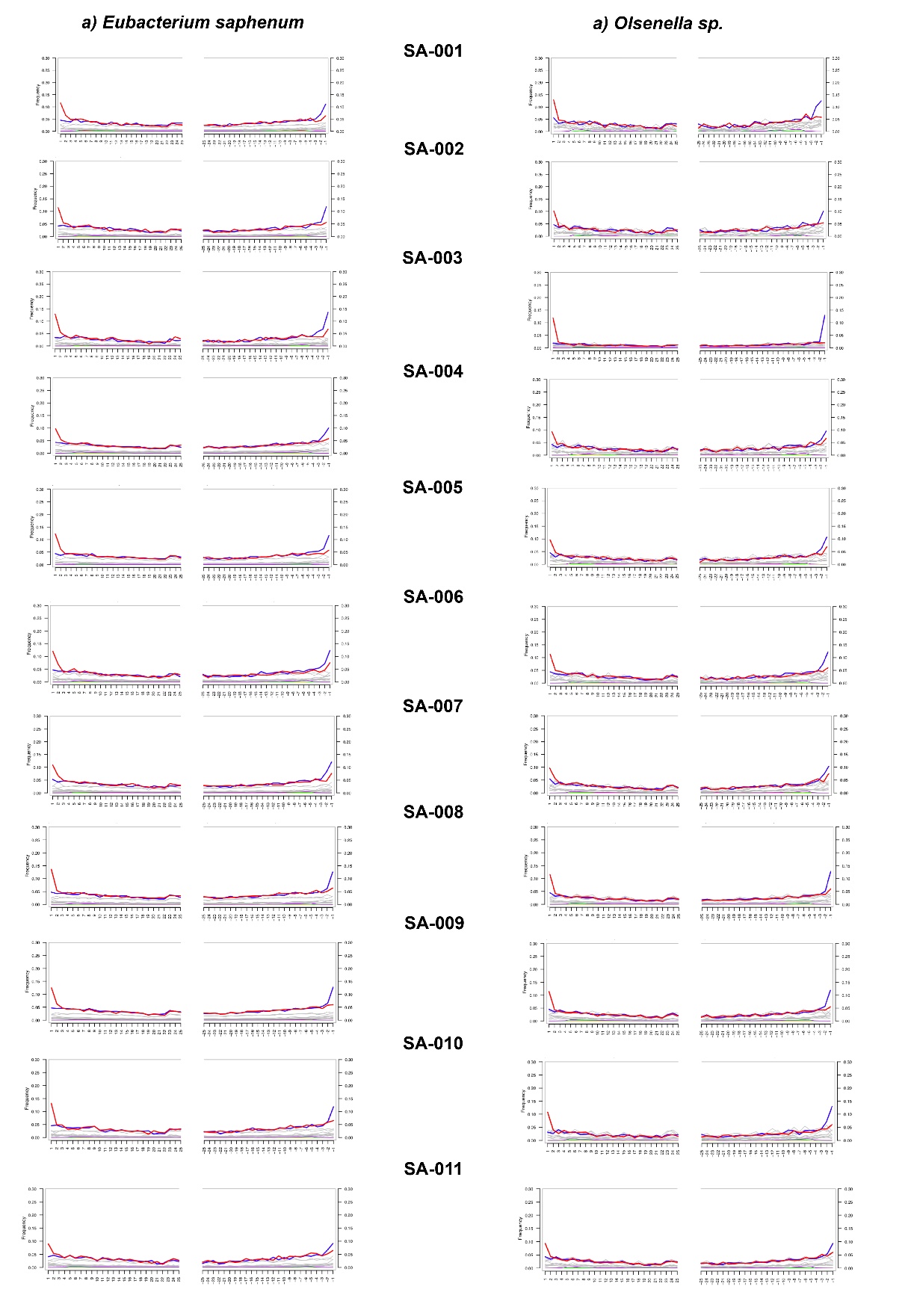


Supplementary Figure 4A-C: Damage plots generated using MapDamage for the keystones identified in Maya dental calculus. Damage patterns are consistent with ancient DNA for the Maya dental calculus keystones


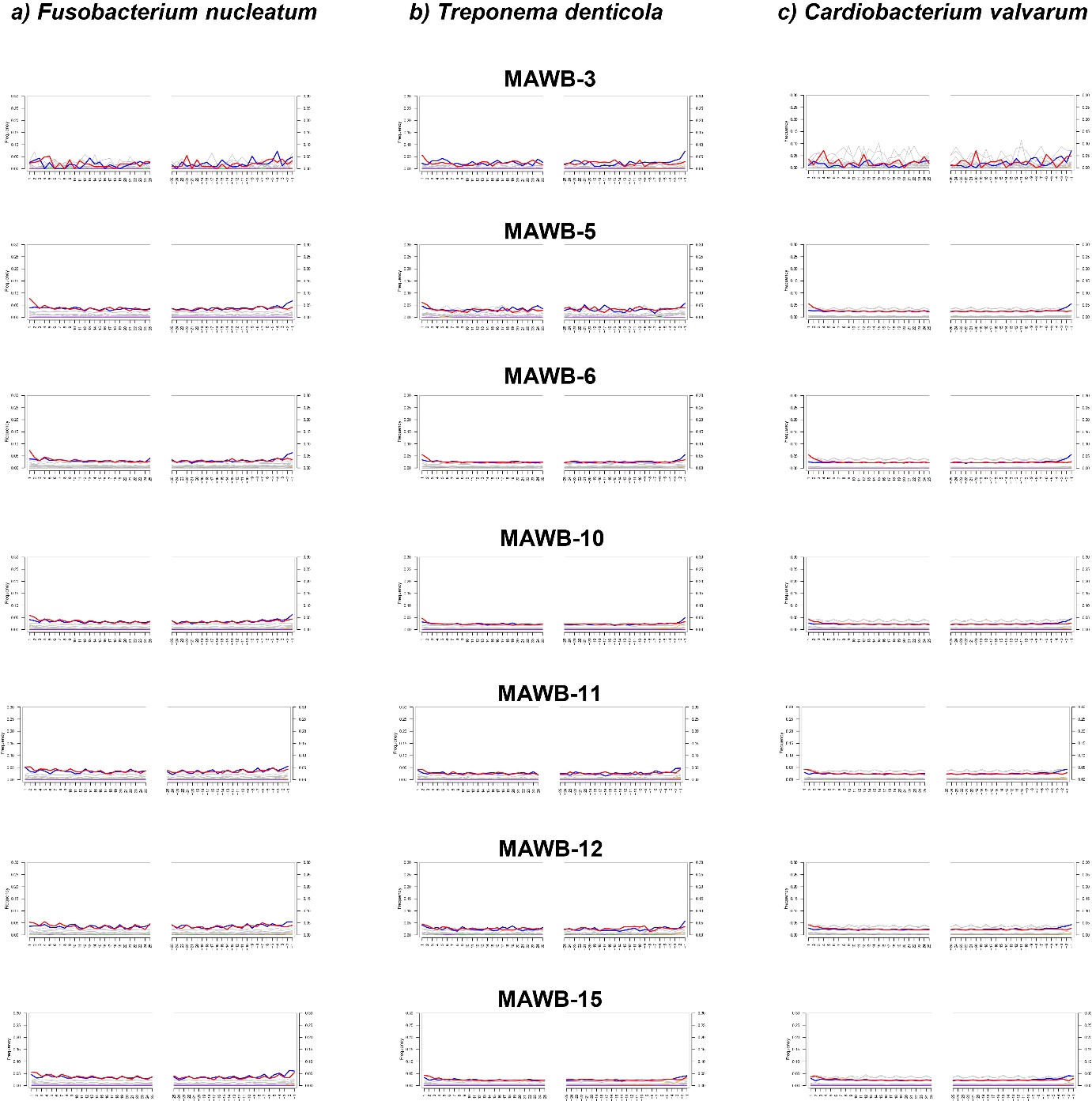


Supplementary Figure 5: Heatmap demonstrating co-occurrence of early colonizing oral taxa and selected periodontitis-associated bacteria (across top of table) with common oral taxa. Networks were generated 100 times and the shade of red represents the number of network iterations that the selected bacteria localize in same cluster as taxa along the y-axis. Cells in gray represent taxa that were not found in each respective dataset. In the Maya and Radcliffe datasets, *A. naeslundii* has a distinct clustering pattern compared to other early colonizing/red complex bacteria, while in the Nuragic population, *S. gordonii* shows a unique clustering pattern.


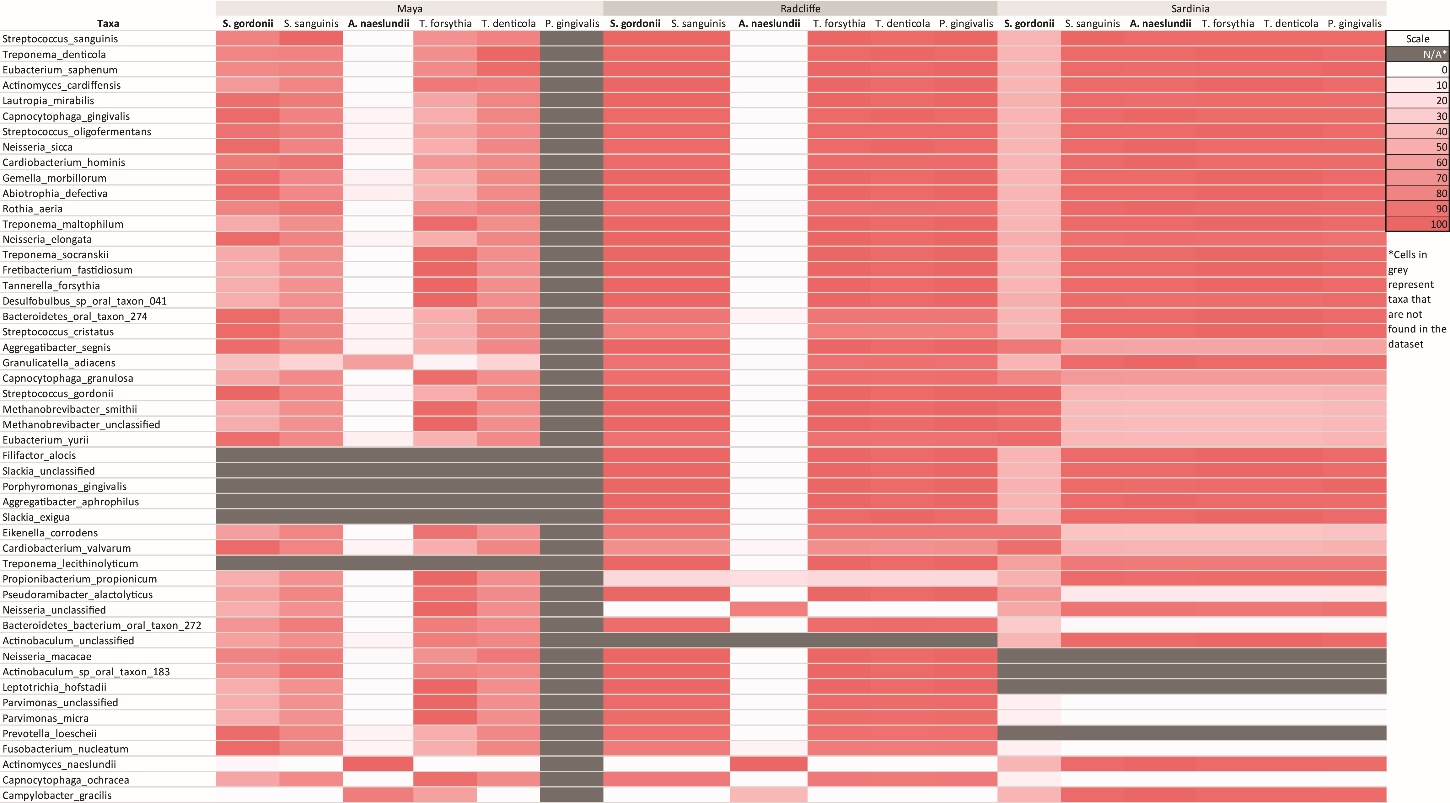


Supplementary Figure 6A-F: Functional diversity between ancient and modern datasets. Significant p-values are given in reference to the Rio Zape coprolites in A-C, while significant p-values are given in reference to the Nuragic dataset in D-F. A-C) Modern non-industrial gut microbiomes are similar to the Rio Zape coprolites, while the modern industrial datasets are more diverse than the coprolites. Increased functional diversity in modern industrial gut microbiomes may be driven by database bias. D) The Nuragic dataset is an outlier for functional diversity compared to the modern and other ancient dental calculus datasets.


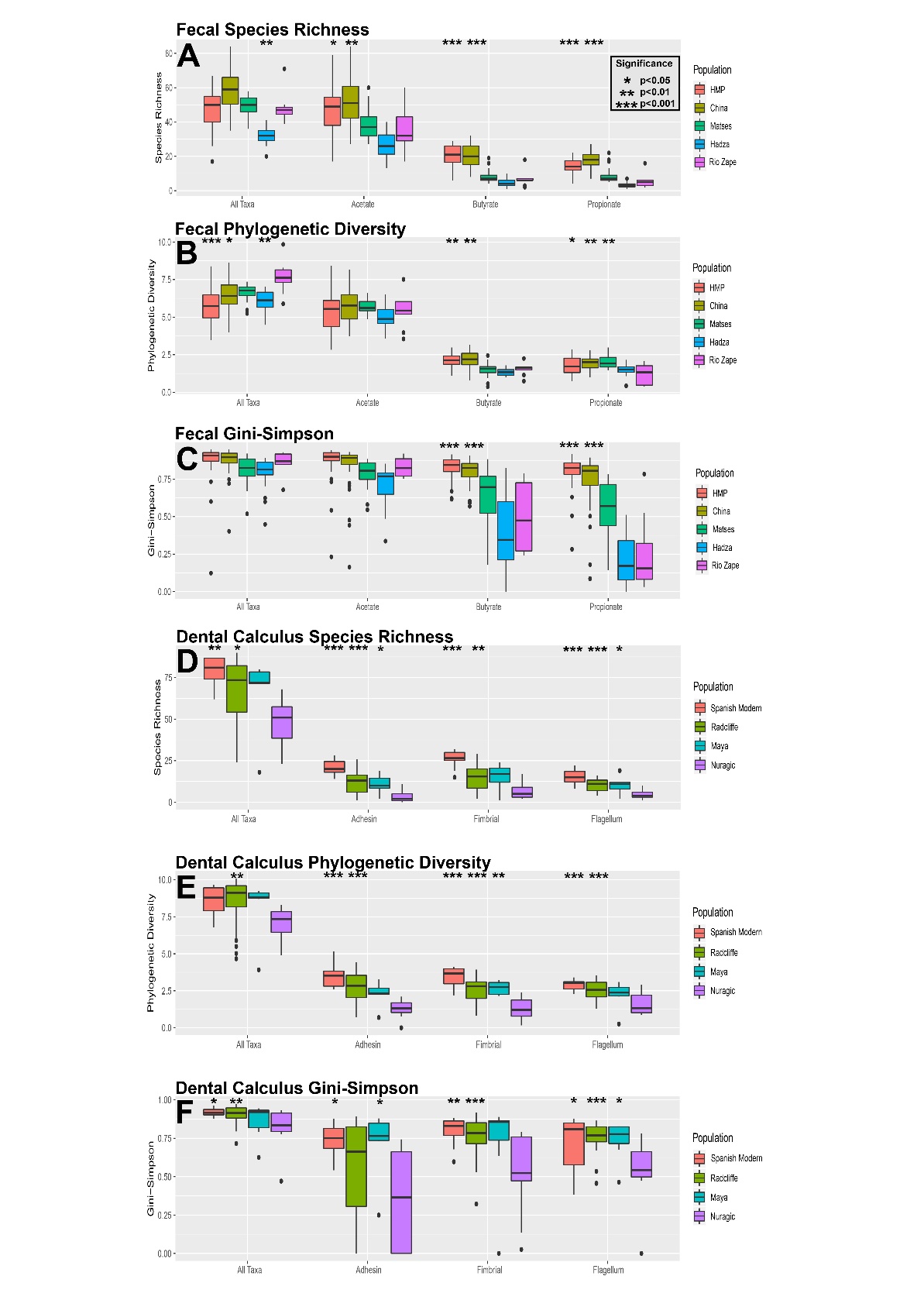


Supplementary Figure 7A-B: Effect of small sample size on network properties for both gut microbiomes and dental calculus. A) The number of clusters increases directly with sample size in both sample types. B)The network distinctness ratio is modularity divided by transitivity and this ratio helps operationalize the interconnectivity of the network. Higher network distinctness is found in networks with clusters and nodes that are distinct from each other. Network distinctness increases with sample size for gut microbiomes but not for ancient dental calculus.


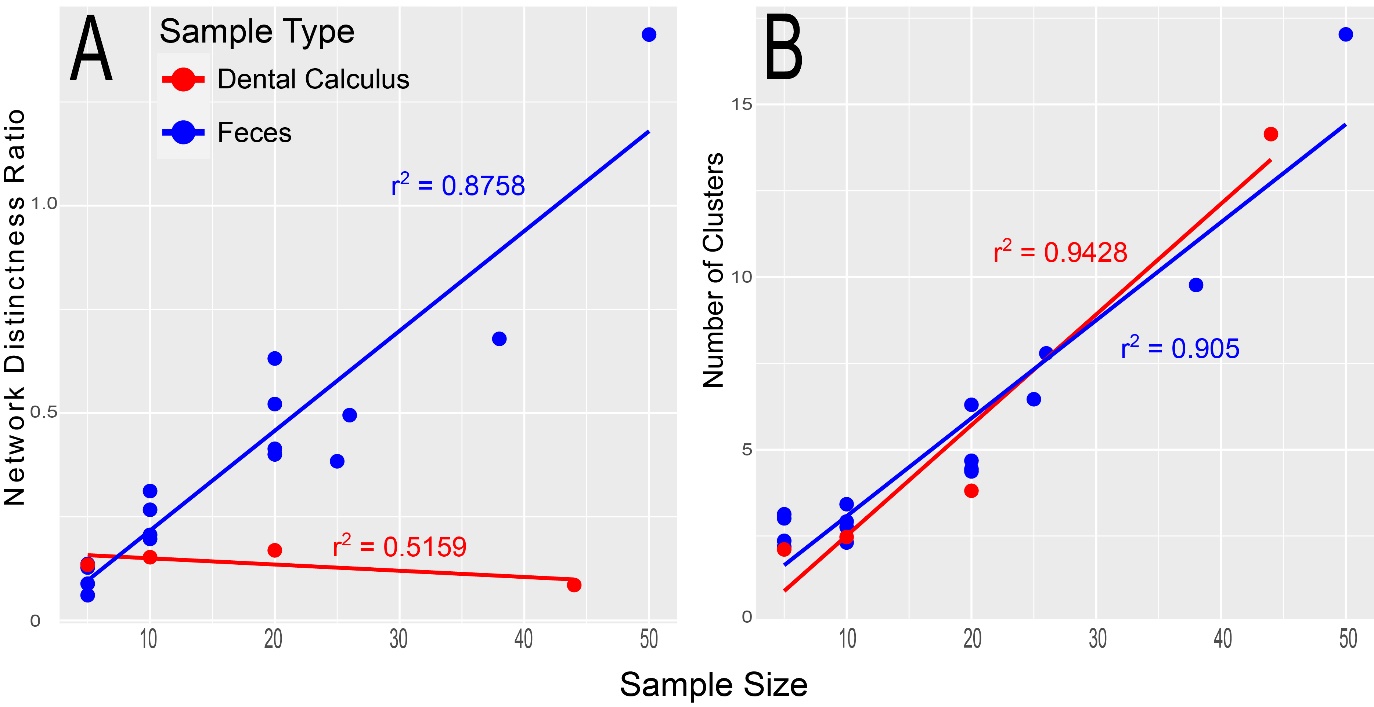


Supplementary Figure 8: Small sample size results in poor recovery of keystones from full dataset. The y-axis represents the percent of keystones from the full dataset that were found in each of the small sample size datasets. Five and Ten sample networks have few keystones matching the keystones from the full dataset.


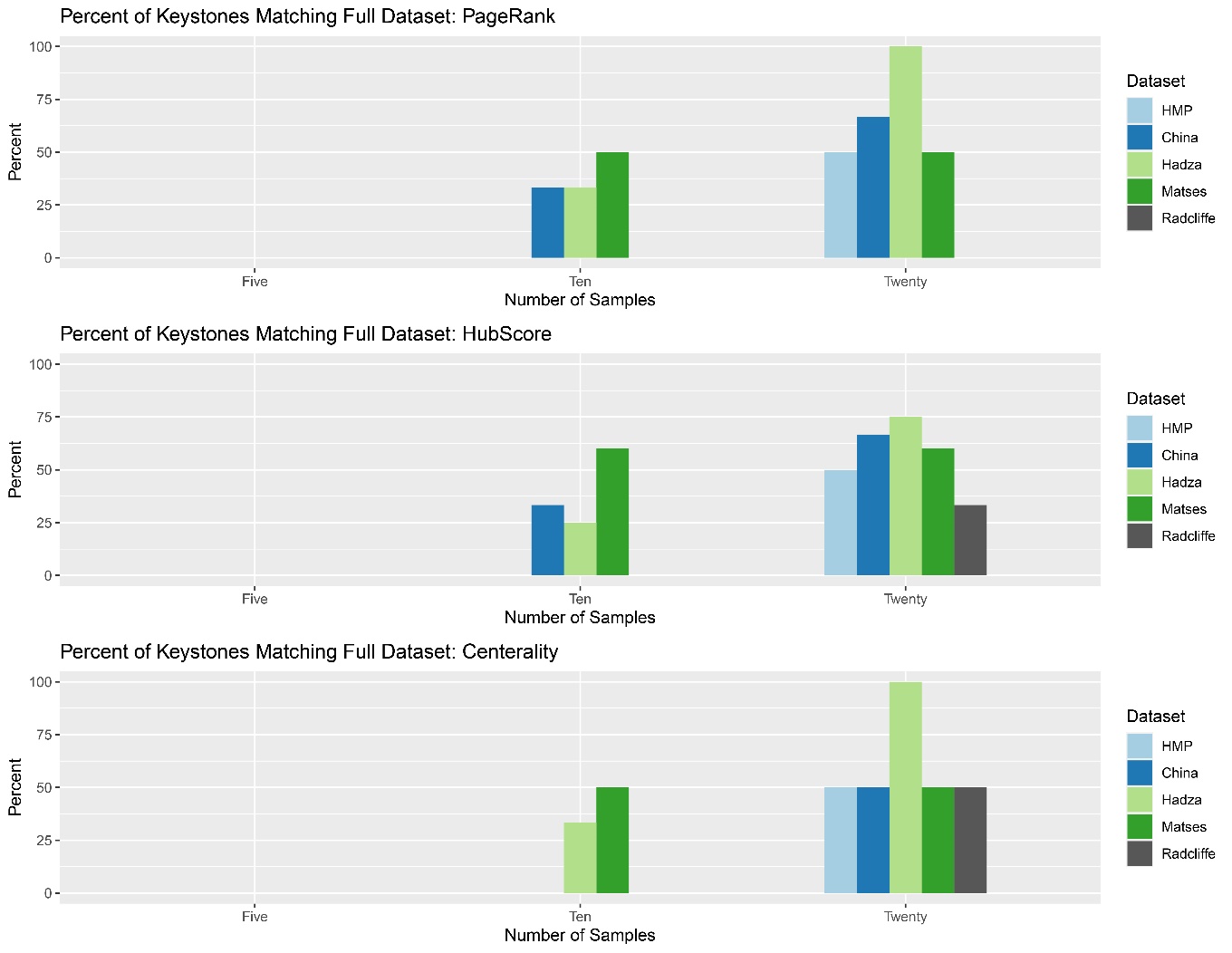


Supplementary Figure 9: A) Visual representation of network properties. Each number represents an individual taxon as a node, each color represents a cluster, each line represents connected nodes, a nodes with thick borders are keystone taxa. B) Network with low modularity (i.e. clusters are highly connected to each other). C) Network with high transitivity (i.e. nodes are connected to other nodes without needing to be connected through a central or keystone taxa). High transitivity and low modularity are often found in the same network.


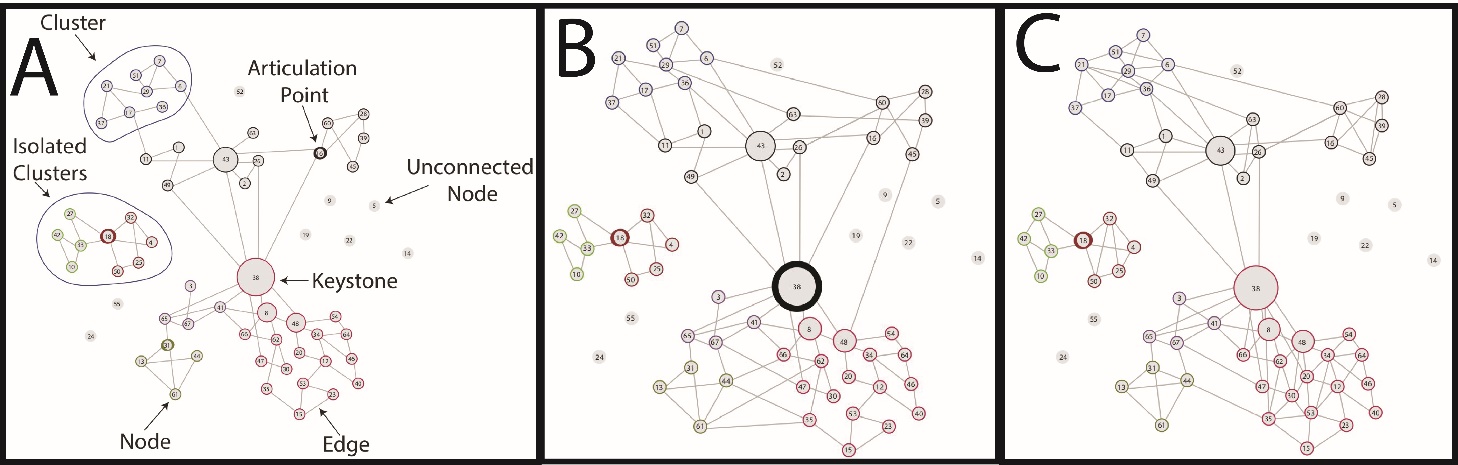


**IV. Supplementary Tables**

Supplementary Table 1, a-d: Top 50 genes by gene abundance from HUMAnN2 for each keystone taxa found in the Rio Zape coprolites. Abundance is gene copies per 1 million gene copies, with the mean value across the dataset reported for each gene. Antibiotic resistance genes are in bold.

| a) Escherichia | |
| --- | --- |
| Gene Name | meanAbund |
| Escherichia coli IMT2125 genomic chromosome, IMT2125 | 157.21948 |
| hypothetical protein | 147.55349 |
| Escherichia coli IMT2125 genomic chromosome, IMT2125\|unclassified | 104.17424 |
| hypothetical protein, partial | 68.28250 |
| Putative membrane protein | 37.22662 |
| Membrane protein | 32.14988 |
| Predicted protein | 28.65321 |
| Escherichia coli 1540 plasmid pIP1206 complete genome | 23.98338 |
| Putative membrane protein (Fragment) | 23.44465 |
| Escherichia coli 1540 plasmid pIP1206 complete genome\|unclassified | 19.47129 |
| Mannitol-1-phosphate 5-dehydrogenase | 18.06854 |
| Conserved domain protein | 14.58802 |
| TTG start codon | 13.55998 |
| Transposase | 10.47736 |
| Transposase family protein | 9.33703 |
| Protein SrnB | 8.47705 |
| Ornithine carbamoyltransferase 1 | 7.97669 |
| RepA3 | 7.73112 |
| Multiple stress resistance protein BhsA domain protein | 7.55945 |
| Thioredoxin reductase | 7.21115 |
| Transposase, IS605 family | 7.18980 |
| PyrBI operon leader peptide | 7.08167 |
| Putative asparagine synthetase B | 6.95930 |
| Acetyltransferase | 6.77352 |
| Enterobactin synthase | 6.35817 |
| Ribonucleoside-diphosphate reductase 1, beta subunit, B2 | 6.34910 |
| MalG gene 3-flanking DNA | 6.09923 |
| Transport of hexuronates | 5.96863 |
| Transcriptional regulator | 5.90242 |
| Hemolysin E, chromosomal domain protein | 5.84491 |
| Cellulose synthase catalytic subunit [UDP-forming] | 5.79237 |
| Glutamyl-tRNA synthetase domain protein | 5.74202 |
| Phage recombination protein Bet (Fragment) | 5.71533 |
| RpmH ribosomal protein L34 | 5.61438 |
| Tryptophan permease | 5.53631 |
| cell division protein FtsL | 5.44006 |
| Phosphatidylserine decarboxylase | 5.41644 |
| Single-stranded DNA-binding protein | 5.38347 |
| Toxin SymE, type I toxin-antitoxin system family protein | 5.30155 |
| Abc transport membrane permease | 5.24969 |
| Putative transposase | 5.21585 |
| Aldo/keto reductase | 5.16524 |
| Protein rof | 5.08851 |
| Putative IS1 encoded protein | 5.05463 |
| Transcription elongation factor | 4.96258 |
| Putative HTH-type transcriptional regulator YneL | 4.96033 |
| Ybl54 | 4.75796 |
| N-acetyl-gamma-glutamyl-phosphate reductase | 4.67759 |
| Sulfate transport system permease protein CysT | 4.64893 |
| Thiamine import ATP-binding protein ThiQ | 4.50032 |
| b) Brachyspira | |
| Gene Name | meanAbund |
| hypothetical protein | 6.42956 |
| TPR domain-containing protein | 6.07162 |
| Lipoprotein | 3.65874 |
| TPR repeat-containing protein | 3.44506 |
| Glycosyl transferase family 2 | 3.23542 |
| Ankyrin repeat-containing protein | 3.08389 |
| Extracellular solute-binding protein, family 5 | 2.91664 |
| **Acriflavin resistance protein** | **2.73465** |
| Pseudouridine synthase | 2.71509 |
| Pyruvate phosphate dikinase | 2.68776 |
| Serpulina hyodysenteriae variable surface protein | 2.48640 |
| Ankyrin | 2.25450 |
| 3-deoxy-7-phosphoheptulonate synthase | 2.18766 |
| Methyltransferase | 2.17242 |
| ABC transporter related protein | 2.13497 |
| Outer membrane protein | 2.01585 |
| Galactose-1-phosphate uridylyltransferase | 2.00448 |
| Appr-1-p processing domain protein | 1.99894 |
| Transporter | 1.75380 |
| Cytidylate kinase | 1.67780 |
| SAM-dependent methyltransferase | 1.67613 |
| hypothetical protein, partial | 1.63529 |
| Radical SAM domain protein | 1.61393 |
| Inner-membrane translocator | 1.55803 |
| Flavodoxin | 1.51300 |
| Extracellular solute-binding protein family 1 | 1.48899 |
| Glycosyltransferase | 1.43515 |
| Peptidase M23 | 1.39032 |
| Tetratricopeptide TPR_2 repeat protein | 1.37358 |
| 50S ribosomal protein L3 (Fragment) | 1.34407 |
| RNA polymerase sigma factor | 1.32794 |
| Phosphopentomutase | 1.30253 |
| Adenine specific DNA methyltransferase | 1.28853 |
| D-3-phosphoglycerate dehydrogenase | 1.28524 |
| Thioredoxin reductase | 1.25088 |
| Putative reductase BN758_00609 | 1.24574 |
| Unclassified | 1.23447 |
| 5-methylcytosine restriction system component-like protein | 1.19024 |
| **MATE efflux family protein** | **1.15107** |
| Uridine phosphorylase | 1.14636 |
| 50S ribosomal protein L1 | 1.14591 |
| 50S ribosomal protein L11 | 1.09380 |
| CheW protein | 1.09207 |
| N-acetylmuramoyl-L-alanine amidase | 1.08677 |
| N-acylglucosamine 2-epimerase | 1.07755 |
| Chemotaxis protein methyltransferase CheR | 1.06766 |
| TatD protein | 1.06152 |
| Carbohydrate kinase, PfkB family | 1.04430 |
| Putative K(+)-stimulated pyrophosphate-energized sodium pump | 1.03691 |
| Methyltransferase type 11 | 1.02666 |
| c) Eubacterium biforme | |
| Gene Name | meanAbund |
| Transposase | 24.28352 |
| Putative transposase DNA-binding domain protein (Fragment) | 20.74955 |
| Transposase-like protein | 13.41998 |
| ABC transporter, ATP-binding protein | 12.67177 |
| **MATE efflux family protein** | **12.28083** |
| 50S ribosomal protein L36 | 10.65286 |
| 50S ribosomal protein L34 | 10.16945 |
| Transposase, IS116/IS110/IS902 family | 8.23550 |
| ATP synthase subunit c | 8.19592 |
| ABC transporter, substrate-binding protein, family 5 | 8.06157 |
| HAD hydrolase, family IA, variant 3 | 7.81502 |
| 30S ribosomal protein S13 | 7.02504 |
| ABC transporter permease protein | 6.96366 |
| 30S ribosomal protein S11 | 6.76403 |
| Transcriptional regulator, TetR family | 6.07580 |
| ATP synthase subunit b | 5.89358 |
| 30S ribosomal protein S14 type Z | 5.89029 |
| Cof-like hydrolase | 5.84100 |
| ATPase/histidine kinase/DNA gyrase B/HSP90 domain protein | 5.83098 |
| Pseudouridine synthase | 5.66666 |
| Single-stranded DNA-binding protein | 5.65279 |
| Amidohydrolase | 5.44604 |
| ATP synthase gamma chain | 5.44145 |
| Addiction module toxin, RelE/StbE family | 5.37597 |
| ATP synthase epsilon chain | 5.36676 |
| 50S ribosomal protein L32 | 5.34519 |
| ATP-dependent zinc metalloprotease FtsH | 5.01661 |
| Diguanylate cyclase (GGDEF) domain protein | 4.97768 |
| DNA-directed RNA polymerase subunit alpha | 4.97711 |
| Amidophosphoribosyltransferase | 4.97575 |
| Transcriptional regulator | 4.92216 |
| 50S ribosomal protein L29 | 4.77886 |
| Elongation factor P | 4.72858 |
| PTS family mannose porter, IIC component | 4.66532 |
| SIS domain protein | 4.66105 |
| 30S ribosomal protein S18 | 4.61437 |
| Transporter | 4.60889 |
| UDP-glucose 4-epimerase | 4.60178 |
| 50S ribosomal protein L33 1 | 4.60120 |
| IS66 family element, transposase | 4.59927 |
| 50S ribosomal protein L31 | 4.52545 |
| 30S ribosomal protein S10 | 4.46549 |
| ATP synthase subunit beta | 4.44541 |
| Peptide deformylase | 4.43869 |
| Putative transposase | 4.36506 |
| PTS system mannose/fructose/sorbose family IIB component | 4.34517 |
| Transcriptional regulator, XRE family | 4.34135 |
| Thioredoxin | 4.32730 |
| Transcriptional regulator, MarR family | 4.32331 |
| Serine carboxypeptidase | 4.31232 |
| d) Phascolarctobacterium succinatutens | |
| Gene Name | meanAbund |
| 50S ribosomal protein L33 1 | 7.80331 |
| DNA-binding helix-turn-helix protein | 6.50082 |
| ATPase/histidine kinase/DNA gyrase B/HSP90 domain protein | 6.17674 |
| Transporter, DASS family | 5.64666 |
| 50S ribosomal protein L30 | 5.55721 |
| Periplasmic binding protein | 4.91736 |
| ABC transporter ATP-binding protein | 4.70193 |
| 4Fe-4S binding domain protein | 4.66279 |
| **MATE efflux family protein** | **4.64157** |
| Elongation factor Tu, apicoplast | 4.59695 |
| Pyridine nucleotide-disulfide oxidoreductase | 3.82089 |
| Nucleoside diphosphate kinase | 3.78357 |
| FAD linked oxidase domain protein | 3.47685 |
| Peptidyl-prolyl cis-trans isomerase | 3.41422 |
| F420-non-reducing hydrogenase iron-sulfur subunit D | 3.36498 |
| 30S ribosomal protein S18 | 3.30487 |
| Oxidoreductase, nitrogenase component 1 | 3.09705 |
| ABC polar amino acid transporter | 3.01383 |
| Outer membrane protein | 2.97801 |
| Transposase | 2.96064 |
| Cobalt transporter | 2.89418 |
| Lipoprotein | 2.89072 |
| 50S ribosomal protein L21 | 2.84896 |
| Cysteine--tRNA ligase | 2.84868 |
| ATPase | 2.81229 |
| TonB-dependent receptor | 2.69838 |
| Monovalent cation/H+ antiporter subunit B domain protein | 2.69413 |
| 30S ribosomal protein S21 | 2.68228 |
| 30S ribosomal protein S13 | 2.67281 |
| DNA-directed RNA polymerase subunit beta | 2.67030 |
| FAD dependent oxidoreductase | 2.65715 |
| Radical SAM domain protein | 2.65164 |
| 2-nitropropane dioxygenase NPD | 2.64955 |
| 50S ribosomal protein L16, chloroplastic | 2.64650 |
| 50S ribosomal protein L35 | 2.61008 |
| Transporter | 2.60551 |
| 50S ribosomal protein L20 | 2.59246 |
| Transposase (Fragment) | 2.58272 |
| 50S ribosomal protein L29 | 2.56885 |
| RNA binding S1 domain protein | 2.51467 |
| 30S ribosomal protein S9 | 2.48475 |
| Extracellular ligand-binding receptor | 2.47446 |
| 50S ribosomal protein L27 | 2.47359 |
| 30S ribosomal protein S15 | 2.44770 |
| Glutamate--tRNA ligase | 2.43361 |
| **Acriflavin resistance protein** | **2.43237** |
| Response regulator receiver domain protein | 2.40683 |
| Rubrerythrin (RR) | 2.40118 |
| Amidohydrolase | 2.36100 |
| ATP synthase subunit b | 2.35636 |

Supplementary Table 2, a-b: Top 50 genes by gene abundance from HUMAnN2 for each keystone taxa found in the Nuragic dental calculus samples. Abundance is gene copies per 1 million gene copies, with the mean value across the dataset reported for each gene. Antibiotic resistance genes are in bold.

| a) Eubacterium saphenum | |
| --- | --- |
| Gene Name | meanAbund |
| Bacterial surface protein 26-residue PARCEL repeat (3 repeats) | 14.95073 |
| 50S ribosomal protein L31 | 12.79862 |
| YibE/F-like protein | 12.63424 |
| Efflux ABC transporter, permease protein | 11.61885 |
| Flavodoxin | 10.75676 |
| 50S ribosomal protein L34 | 8.60214 |
| TIGR02185 family protein | 8.56613 |
| DNA-damage-inducible protein D family protein | 8.36224 |
| NA+/H+ antiporter NHAC | 8.27645 |
| Elongation factor Tu, apicoplast | 7.88859 |
| Repeat protein | 7.62742 |
| 30S ribosomal protein S21 | 7.57228 |
| Fic family protein | 6.80146 |
| Phenazine biosynthesis protein, PhzF family | 6.67937 |
| CoA-binding domain protein | 6.61519 |
| AMP-binding enzyme | 6.53746 |
| Translation initiation factor IF-1 | 6.49625 |
| Hypothetical bacterial integral membrane protein (Trep_Strep) | 6.44447 |
| 30S ribosomal protein S17 | 6.14516 |
| LPXTG-motif cell wall anchor domain protein | 6.11107 |
| Polysaccharide deacetylase | 6.01976 |
| Bacterial group 2 Ig-like protein | 6.01798 |
| Biotin synthase | 5.97151 |
| 30S ribosomal protein S13 | 5.87702 |
| 50S ribosomal protein L18 | 5.79206 |
| Papain family cysteine protease | 5.78749 |
| 50S ribosomal protein L30 | 5.72232 |
| 50S ribosomal protein L29 | 5.57128 |
| Bacteriocin-associated integral membrane protein | 5.43963 |
| Putative septation protein SpoVG | 5.39673 |
| DNA-binding protein HU | 5.39548 |
| RIP metalloprotease RseP | 5.31936 |
| 50S ribosomal protein L35 | 5.31727 |
| 30S ribosomal protein S7 | 5.26371 |
| 50S ribosomal protein L5 | 5.19890 |
| 30S ribosomal protein S14 type Z | 5.17576 |
| 30S ribosomal protein S8 | 5.16821 |
| Serine-type D-Ala-D-Ala carboxypeptidase | 5.15686 |
| 50S ribosomal protein L14 | 5.06668 |
| Phage major tail protein, phi13 family | 4.75000 |
| Bacteriocin, lactococcin 972 family | 4.70212 |
| Amino acid permease-associated region | 4.63399 |
| FMN-binding domain protein | 4.60785 |
| DNA repair protein RecO | 4.56762 |
| 30S ribosomal protein S18 | 4.52065 |
| 50S ribosomal protein L16 | 4.46216 |
| Tryptophanase | 4.44278 |
| 30S ribosomal protein S15 | 4.38201 |
| 30S ribosomal protein S6 | 4.30991 |
| V-type sodium ATPase K subunit | 4.25547 |
| b) Olsenella | |
| Gene Name | meanAbund |
| DNA-binding helix-turn-helix protein | 26.92384 |
| ABC1 family protein | 14.98862 |
| ABC transporter, ATP-binding protein | 11.84994 |
| ABC-2 family transporter protein | 10.22147 |
| MacB-like periplasmic core domain protein | 10.01913 |
| Transcriptional regulator, DeoR family | 9.55413 |
| Acetolactate synthase, small subunit | 8.56009 |
| Glycoside hydrolase, family 25 | 8.43790 |
| PF14335 domain protein | 8.24566 |
| Transcriptional regulator, ArsR family | 8.03606 |
| Transcriptional regulator, AbrB family | 7.30884 |
| PF13635 domain protein | 7.30504 |
| ATP-dependent DNA helicase RecG C-terminal domain protein | 7.14551 |
| Haloacid dehalogenase-like hydrolase | 6.91339 |
| Ketopantoate reductase ApbA/PanE domain protein | 6.72580 |
| Nucleotidyl transferase, PF08843 domain protein | 6.65711 |
| Haloacid dehalogenase-like hydrolase domain protein | 6.63774 |
| Site-specific recombinase, phage integrase domain protein | 6.41275 |
| Toxin-antitoxin system, toxin component, Fic domain protein | 6.29031 |
| Site-specific recombinase, phage integrase family | 6.28408 |
| GHKL domain protein | 6.26230 |
| HAD-superfamily hydrolase, subfamily IIB | 6.10579 |
| Alpha/beta hydrolase fold-3 domain protein | 5.97270 |
| DNA methylase family protein | 5.73054 |
| Cinnamoyl ester hydrolase | 5.65442 |
| HAD hydrolase, family IA, variant 3 | 5.42257 |
| Putative major cell-binding factor | 5.02274 |
| Histidinol phosphate phosphatase HisJ family | 4.97715 |
| Cell division protein FtsZ | 4.96390 |
| Zinc-finger of transposase IS204/IS1001/IS1096/IS1165 (Fragment) | 4.89528 |
| Integral membrane sensor signal transduction histidine kinase | 4.80561 |
| 3-isopropylmalate dehydrogenase | 4.66355 |
| SCP-2 sterol transfer family protein | 4.55784 |
| Short-chain dehydrogenase/reductase SDR | 4.53504 |
| Acetylornithine aminotransferase | 4.38097 |
| Virulence activator alpha C-terminal family protein | 4.35334 |
| Acetylglutamate kinase | 4.32056 |
| Calcineurin-like phosphoesterase family protein | 4.26203 |
| Transposase domain protein (Fragment) | 4.17989 |
| Fic/DOC family protein | 4.07258 |
| TatD-related deoxyribonuclease | 4.06937 |
| Galactokinase galactose-binding signature | 3.99070 |
| Peptidase, S9A/B/C family, catalytic domain protein | 3.88265 |
| Basic membrane domain protein | 3.87643 |
| PF06115 domain protein | 3.86580 |
| Small molecule-binding regulator domain protein | 3.85141 |
| PF14014 family protein | 3.80053 |
| Sortase, SrtB family | 3.73665 |
| ATPase | 3.73579 |
| N-acetylmuramoyl-L-alanine amidase domain protein | 3.70939 |

Supplementary Table 3, a-c: Top 50 genes by gene abundance from HUMAnN2 for each keystone taxa found in the Mayan dental calculus samples. Abundance is gene copies per 1 million gene copies, with the mean value across the dataset reported for each gene. Antibiotic resistance genes are in bold

| Fusobacterium nucleatum | |
| --- | --- |
| Gene Name | meanAbund |
| Cell wall-associated hydrolase | 79.57285 |
| Transposase | 20.11884153 |
| Hypothetical cytosolic protein | 9.835568 |
| Flavodoxin | 5.583699738 |
| Transporter | 5.241214675 |
| Transposase, IS605 OrfB family | 5.16738375 |
| Integral membrane protein | 4.89230775 |
| Transposase, IS605 OrfB family, central region | 4.47566375 |
| Possible transcriptional regulator | 3.867855963 |
| Hypothetical Cytosolic Protein | 3.838350738 |
| **MATE efflux family protein** | **3.603400888** |
| ISChy9, transposase OrfB | 3.3649925 |
| Transcriptional regulator | 3.321478463 |
| Peptidyl-prolyl cis-trans isomerase | 3.317424 |
| Methyltransferase | 3.10504935 |
| Ethanolamine utilization protein | 2.8974015 |
| Transposase-like protein B | 2.85549875 |
| MORN repeat protein | 2.7722654 |
| Cysteine synthase | 2.760272625 |
| Tetratricopeptide repeat family protein | 2.759906438 |
| Lipoprotein | 2.634973 |
| Predicted protein | 2.536824525 |
| Transcriptional regulator, TetR family | 2.480689325 |
| Acetyltransferase | 2.47860535 |
| Radical SAM domain protein | 2.45034175 |
| Outer membrane protein | 2.432701088 |
| Possible transposase | 2.364140413 |
| Pseudouridine synthase | 2.265692875 |
| Hemolysin | 2.24385648 |
| Transposase IS116/IS110/IS902 family protein | 2.22705 |
| Thioredoxin reductase | 2.127039413 |
| IS1296 transposase protein B | 2.076046 |
| Conserved protein | 2.015840725 |
| 50S ribosomal protein L34 | 1.992396 |
| VWA containing CoxE family protein | 1.98906125 |
| DNA-directed RNA polymerase subunit beta | 1.96381375 |
| NA+/H+ antiporter NHAC | 1.935227938 |
| Zinc finger SWIM domain protein | 1.8977675 |
| ATPase | 1.85072545 |
| Thymidylate synthase | 1.84568525 |
| Amidohydrolase | 1.83815185 |
| Transcriptional regulator, DeoR family | 1.820777925 |
| Possible tyrosine transporter P-protein | 1.76818 |
| Anthranilate synthase component II | 1.7671905 |
| GCN5-related N-acetyltransferase | 1.761517788 |
| RfaE bifunctional protein | 1.731037375 |
| Cobalt-precorrin-4 C(11)-methyltransferase | 1.72463875 |
| Guanine-hypoxanthine permease | 1.711039625 |
| Manganese-binding protein | 1.694659 |
| Cobyric acid synthase | 1.676362788 |
| Treponema denticola | |
| Gene Name | meanAbund |
| Transcriptional regulator, TetR family | 6.25098 |
| ABC transporter, ATP-binding protein | 5.42128 |
| Ankyrin repeat protein | 3.06271 |
| Site-specific recombinases, DNA invertase Pin homologs | 3.01433 |
| Lipoprotein | 2.40664 |
| Thioredoxin | 2.28700 |
| Methyl-accepting chemotaxis protein | 2.18394 |
| ABC transporter ATP-binding protein | 1.74008 |
| **MATE efflux family protein** | **1.42679** |
| Glutathione peroxidase | 1.38907 |
| Membrane protein, putative | 1.35796 |
| ABC transporter ATP-binding protein/permease | 1.29008 |
| DNA mismatch endonuclease Vsr | 1.05253 |
| Xenobiotic-transporting ATPase | 0.99221 |
| Ribonuclease VapC | 0.95592 |
| TPR protein | 0.94999 |
| Oligopeptide/dipeptide ABC transporter, ATP-binding protein | 0.91104 |
| ABC-type multidrug transport system, ATPase and permease component | 0.88981 |
| Diguanylate cyclase (GGDEF) domain-containing protein | 0.84254 |
| Pseudouridine synthase | 0.84222 |
| YcfA family protein | 0.81817 |
| 50S ribosomal protein L32 | 0.76883 |
| ABC transporter related protein | 0.76582 |
| ABC transporter | 0.74570 |
| RluA family pseudouridine synthase | 0.73355 |
| Heavy metal translocating P-type ATPase | 0.73148 |
| Metallo-beta-lactamase family protein | 0.72542 |
| Histidine kinase | 0.71756 |
| Conserved domain protein | 0.71313 |
| Addiction module antitoxin, RelB/DinJ family | 0.67645 |
| RNA polymerase sigma factor | 0.63557 |
| L-lactate dehydrogenase | 0.62536 |
| RelB/DinJ family addiction module antitoxin | 0.59393 |
| Integrase catalytic region | 0.58639 |
| Chorismate mutase | 0.58630 |
| Oligopeptide transport ATP-binding protein AppD | 0.58623 |
| RpiB/LacA/LacB family sugar-phosphate isomerase | 0.58315 |
| Possible dnaK suppressor | 0.57961 |
| Cobalt transport protein | 0.57135 |
| LysM/M23/M37 peptidase | 0.54945 |
| ATPase AAA | 0.54873 |
| Prevent-host-death family protein | 0.54788 |
| Flagellar hook-basal body complex protein FliE | 0.54580 |
| **MATE family transporter** | **0.53897** |
| Signal peptidase I | 0.53443 |
| Peptidase M42 family protein | 0.53376 |
| RNA methyltransferase | 0.53039 |
| Peptidyl-prolyl cis-trans isomerase | 0.52088 |
| RHS repeat-associated core domain-containing protein | 0.51748 |
| DNA polymerase III | 0.50657 |
| Cardiobacterium valvarum | |
| Gene Name | meanAbund |
| Transposase | 53.58075 |
| Sel1 repeat protein | 30.17798 |
| Helix-turn-helix domain of resolvase (Fragment) | 21.38863 |
| IS1480b transposase | 15.72224 |
| Tetratricopeptide repeat protein | 13.64956 |
| Acetyltransferase, GNAT family | 13.18181 |
| IS5 family transposase,Transposase DDE domain | 12.22235 |
| Tat pathway signal sequence domain protein | 12.04198 |
| Peptidyl-prolyl cis-trans isomerase | 11.38757 |
| Ser/Thr phosphatase family protein | 10.17345 |
| ATPase/histidine kinase/DNA gyrase B/HSP90 domain protein | 7.98308 |
| Membrane protein | 7.92403 |
| ABC transporter, ATP-binding protein | 7.59536 |
| Acyl carrier protein | 7.50314 |
| Addiction module antitoxin, RelB/DinJ family | 7.48685 |
| SMI1 / KNR4 family protein | 7.48015 |
| Pseudouridine synthase | 6.95228 |
| DNA-binding helix-turn-helix protein | 6.69018 |
| Transglycosylase SLT domain protein | 6.68673 |
| OmpA family protein | 6.57143 |
| **Efflux transporter, RND family, MFP subunit** | **6.56882** |
| NlpC/P60 family protein | 6.33083 |
| Glyoxalase/bleomycin resistance protein/dioxygenase | 6.22122 |
| Transcriptional regulator, AraC family | 6.22077 |
| Peptidase, M48 family | 6.19822 |
| Acyltransferase | 6.14974 |
| HAD hydrolase, family IA, variant 3 | 5.91526 |
| S4 domain protein | 5.88368 |
| Phosphoglycerate mutase | 5.85409 |
| CRISPR-associated endoribonuclease Cas2 | 5.84203 |
| Transcriptional regulator, DeoR family | 5.80208 |
| Spermidine N(1)-acetyltransferase | 5.77363 |
| NAD dependent epimerase/dehydratase family protein | 5.65739 |
| Hydrolase, TatD family | 5.64212 |
| DnaJ domain protein | 5.63168 |
| Cof-like hydrolase | 5.48318 |
| Response regulator receiver domain protein | 5.42215 |
| LysR substrate binding domain protein | 5.37501 |
| ABC transporter ATP-binding protein | 5.16074 |
| Endonuclease III | 5.10799 |
| Band 7 protein | 5.08845 |
| Two component transcriptional regulator, winged helix family | 5.02484 |
| Exodeoxyribonuclease III | 4.92089 |
| ADP-ribose pyrophosphatase | 4.91616 |
| Peptidase propeptide and YPEB domain protein | 4.86149 |
| Carbamate kinase | 4.83808 |
| Peptidase, S54 family | 4.73912 |
| 3-oxoacyl-[acyl-carrier-protein] reductase FabG | 4.65901 |
| ABC-2 type transporter | 4.63549 |
| Bacterioferritin | 4.62869 |

Supplementary Table 4, a-b: Top 50 genes by gene abundance from HUMAnN2 for each keystone taxa found in the Radcliffe dental calculus samples. Abundance is gene copies per 1 million gene copies, with the mean value across the dataset reported for each gene. Antibiotic resistance genes are in bold

| Treponema socranskii | |
| --- | --- |
| Gene Name | meanAbund |
| ABC-type transporter, integral membrane subunit. | 18.17577 |
| Binding-protein-dependent transport systems inner membrane component. | 16.40026 |
| ABC transporter, permease protein. | 15.47453 |
| DNA-binding helix-turn-helix protein. | 15.08302 |
| ABC transporter, solute-binding protein. | 14.59235 |
| Flavocytochrome c. | 12.64163 |
| Transcriptional regulator, TetR family. | 11.83416 |
| Tetratricopeptide repeat protein. | 11.82341 |
| **MATE efflux family protein.** | **11.47407** |
| Extracellular solute-binding protein family 1. | 10.95863 |
| Pseudouridine synthase. | 10.13271 |
| ABC transporter, ATP-binding protein. | 8.95850 |
| ABC transporter related protein. | 8.76818 |
| Methyltransferase domain protein. | 8.17172 |
| Methyl-accepting chemotaxis protein. | 7.97500 |
| Ferredoxin. | 7.59735 |
| Putative lipoprotein. | 7.34340 |
| Tripartite tricarboxylate transporter TctB family protein. | 7.15727 |
| Transcriptional regulator, DeoR family. | 7.02176 |
| Inner-membrane translocator. | 6.78443 |
| Glycosyl transferase group 1. | 6.75112 |
| Transcriptional regulator, LacI family. | 6.60438 |
| Transcriptional regulator. | 6.33526 |
| TRAP transporter, DctQ-like membrane protein. | 6.03125 |
| Response regulator receiver domain protein. | 5.98242 |
| Tripartite tricarboxylate transporter family receptor. | 5.89056 |
| Ribose import ATP-binding protein RbsA. | 5.54881 |
| FMN-binding domain protein. | 5.25295 |
| RNA polymerase sigma factor. | 5.05100 |
| ABC transporter ATP-binding protein. | 4.98376 |
| Tetratricopeptide TPR_2 repeat-containing protein. | 4.96469 |
| Beta-lactamase domain protein. | 4.94554 |
| Phosphonate-transporting ATPase. | 4.78914 |
| DEAD/DEAH box helicase domain protein. | 4.78738 |
| Elongation factor G. | 4.68901 |
| Ketose-bisphosphate aldolase. | 4.67159 |
| Amidohydrolase family protein. | 4.60343 |
| AAA domain protein. | 4.60341 |
| Na+/H+ antiporter family protein. | 4.58608 |
| PF03382 family protein. | 4.53589 |
| ABC transporter permease protein. | 4.47630 |
| OmpA family protein. | 4.46184 |
| HRDC domain protein. | 4.45457 |
| tRNA/rRNA methyltransferase (SpoU). | 4.44506 |
| Seryl-tRNA synthetase. | 4.43007 |
| ABC-3 protein. | 4.30286 |
| Transposase IS4 family protein. | 4.29741 |
| Acyl carrier protein. | 4.19149 |
| Peptidase, M23 family. | 4.18883 |
| PF04365 family protein. | 4.16101 |
| Tannerella forsythia | |
| Gene Name | meanAbund |
| Putative lipoprotein | 180.16561 |
| TonB-linked outer membrane protein, SusC/RagA family | 90.41168 |
| SusD family protein | 75.76149 |
| TonB-dependent receptor | 73.71884 |
| Tetratricopeptide repeat protein | 68.89489 |
| Sigma factor regulatory protein, FecR/PupR family | 63.44114 |
| Transposase, IS116/IS110/IS902 family | 62.89150 |
| Bacterial group 2 Ig-like protein | 53.26293 |
| RNA polymerase sigma-70 factor | 52.97999 |
| Radical SAM domain protein | 49.85693 |
| Glycosyltransferase, group 1 family protein | 49.10539 |
| Putative membrane protein | 43.28465 |
| ABC transporter ATP-binding protein | 35.57676 |
| Peptidyl-prolyl cis-trans isomerase | 32.97369 |
| Efflux ABC transporter, permease protein | 32.82248 |
| Peptidase, S41 family | 30.92127 |
| Transposase, IS4 family | 30.56842 |
| ATPase/histidine kinase/DNA gyrase B/HSP90 domain protein | 30.47830 |
| Methyltransferase domain protein | 29.27074 |
| Transposase | 27.68744 |
| Outer membrane efflux protein | 25.75341 |
| Response regulator receiver domain protein | 24.45379 |
| Transcriptional regulator, LuxR family | 23.92552 |
| Sigma-70 region 2 | 23.36289 |
| Tat pathway signal sequence domain protein | 21.25475 |
| **Efflux transporter, RND family, MFP subunit** | **21.22168** |
| Transcriptional regulator, TetR family | 20.84302 |
| Outer membrane protein | 20.78598 |
| **MATE efflux family protein** | **20.08566** |
| Acyl carrier protein | 19.64659 |
| Signal peptidase I | 19.55459 |
| Arylsulfatase | 18.74932 |
| Peptidase, S9A/B/C family, catalytic domain protein | 18.31288 |
| Endonuclease/exonuclease/phosphatase family protein | 17.78770 |
| Acyltransferase | 17.70165 |
| Polysaccharide biosynthesis protein | 17.67078 |
| Antioxidant, AhpC/TSA family | 17.18099 |
| PAP2 family protein | 16.63161 |
| RHS repeat-associated core domain protein | 16.38480 |
| Polysaccharide deacetylase | 15.64873 |
| TonB-dependent receptor plug domain protein | 15.60416 |
| Repeat protein | 15.01718 |
| RNA polymerase sigma factor, sigma-70 family | 14.87160 |
| ATPase | 14.72085 |
| TIGR01200 family protein | 14.42236 |
| Pseudouridine synthase | 14.01312 |
| Glycosyltransferase, group 2 family protein | 13.85109 |
| PepSY domain protein | 13.59936 |
| Ser/Thr phosphatase family protein | 13.44160 |
| Sporulation and cell division repeat protein | 13.28620 |

Supplementary Table 5: Mean total clusters increase with sample size, regardless of sample type.

| Sample Type | Population | Mean # of Clusters: 5 Samples | Mean # of Clusters: 10 Samples | Mean # of Clusters: 20 Samples | Mean # of Clusters: All Samples |
| --- | --- | --- | --- | --- | --- |
| Feces | Matses (n = 25) | 2.19 (sd = 0.51) | 2.31 (sd = 0.73) | 4.37 (sd = 1.35) | 6.46 (sd = 1.50) |
|  | Hadza ( n= 26) | 3.01 (sd = 1.03) | 2.92 (sd = 0.51) | 6.3 (sd = 1.69) | 7.79 (sd = 1.65) |
|  | China (n = 38) | 2.36 (sd = 1.06) | 2.76 (sd = 1.18) | 4.44 (sd = 0.90) | 9.77 (sd = 3.29) |
|  | Hmp (n = 50) | 3.13 (sd = 0.66) | 3.42 (sd = 0.95) | 4.68 (sd = 1.38) | 17.03 (sd = 3.23) |
| Dental Calculus | Radcliffe (n = 44) | 2.11 (sd = 0.33) | 2.47 (sd = 0.80) | 3.81 (sd = 0.62) | 14.14 (sd = 3.30) |

Supplementary Table 6: Keystone found in small sample size simulations do not match the keystones identified at the full sample size. Values in each table represent the number of keystones found in the small sample size datasets that were also found in the full dataset for each method of identifying keystone taxa.

| Page Rank | | | | | |
| --- | --- | --- | --- | --- | --- |
|  | Population | n = 5 | n = 10 | n = 20 | Full Dataset |
| Feces | Matses (n = 25) | 0 | 2 | 2 | 4 |
|  | Hadza ( n= 26) | 0 | 1 | 3 | 3 |
|  | China (n = 38) | 0 | 1 | 2 | 3 |
|  | Hmp (n = 50) | 0 | 0 | 1 | 2 |
| Dental Calculus | Radcliffe (n = 44) | 0 | 0 | 0 | 1 |
| Hub Score | | | | | |
|  | Population | n = 5 | n = 10 | n = 20 | Full Dataset |
| Feces | Matses (n = 25) | 0 | 3 | 3 | 5 |
|  | Hadza ( n= 26) | 0 | 1 | 3 | 4 |
|  | China (n = 38) | 0 | 1 | 2 | 3 |
|  | Hmp (n = 50) | 0 | 0 | 1 | 2 |
| Dental Calculus | Radcliffe (n = 44) | 0 | 0 | 1 | 3 |
| Closeness Centrality | | | | | |
|  | Population | n = 5 | n = 10 | n = 20 | Full Dataset |
| Feces | Matses (n = 25) | 0 | 2 | 2 | 4 |
|  | Hadza ( n= 26) | 0 | 1 | 3 | 3 |
|  | China (n = 38) | 0 | 0 | 1 | 2 |
|  | Hmp (n = 50) | 0 | 0 | 1 | 2 |
| Dental Calculus | Radcliffe (n = 44) | 0 | 0 | 1 | 2 |

Supplementary Table 7: Archaeological and anatomical context for samples corresponding to novel dental calculus data generated in this study.

| **LMAMR Sample ID** | **Population** | **Archaeological ID** | **Individual ID** | **Archaeological Site** |
| --- | --- | --- | --- | --- |
| SA-001 | Nuragic (Sardinia) | LMC1: MSAE 6506 | 6506 | Lu Maccioni |
| SA-002 | Nuragic (Sardinia) | LMC2: MSAE 6525 | 6525 | Lu Maccioni |
| SA-003 | Nuragic (Sardinia) | LMC3: MSAE 6515 | 6515 | Lu Maccioni |
| SA-004 | Nuragic (Sardinia) | LMC4: MSAE 6507 | 6507 | Lu Maccioni |
| SA-005 | Nuragic (Sardinia) | CPP1: MSAE 6151 | 6151 | Capo Pecora |
| SA-006 | Nuragic (Sardinia) | CPP2: MSAE 6153 | 6153 | Capo Pecora |
| SA-007 | Nuragic (Sardinia) | CPP3: MSAE 6176 | 6176 | Capo Pecora |
| SA-008 | Nuragic (Sardinia) | CPP4: MSAE 6120 | 6120 | Capo Pecora |
| SA-009 | Nuragic (Sardinia) | SRD1: MSAE 6626 | 6626 | Perdalba |
| SA-010 | Nuragic (Sardinia) | SRD2: MSAE 6623 | 6623 | Perdalba |
| SA-011 | Nuragic (Sardinia) | SRD3: MSAE 6612 | 6612 | Perdalba |
| MAWB-3 | Maya (Belize) | BRV-CH19 | CH19 | Chan |
| MAWB-5 | Maya (Belize) | BRV-CH6 | CH6 | Chan |
| MAWB-6 | Maya (Belize) | CC-B12 | 1 | Chan Chich |
| MAWB-10 | Maya (Belize) | CC-B14 | 1 | Chan Chich |
| MAWB-11 | Maya (Belize) | CC-B14 | 1 | Chan Chich |
| MAWB-12 | Maya (Belize) | CC-B14 | 1 | Chan Chich |
| MAWB-15 | Maya (Belize) | CC-B14 | 1 | Chan Chich |
| **LMAMR Sample ID** | **Date** | **Radiocarbon or Achaeological** | **Tooth sampled** | **Total Raw Reads** |
| SA-001 | 1126-825 calBCE | Radiocarbon (from the same layer) | Mandibular second left molar | 16,317,989 |
| SA-002 | 1126-825 calBCE | Radiocarbon (from the same layer) | Mandibular second right molar | 12,375,354 |
| SA-003 | 1126-825 calBCE | Radiocarbon (from the same layer) | Mandibular third right molar | 11,376,242 |
| SA-004 | 1126-825 calBCE | Radiocarbon (from the same layer) | Mandibular third left molar | 13,545,219 |
| SA-005 | 1384-936 calBCE | Radiocarbon (from the same layer) | Maxillary left incisor | 15,259,048 |
| SA-006 | 1384-936 calBCE | Radiocarbon (from the same layer) | Maxillary third second molar | 11,169,001 |
| SA-007 | 1384-936 calBCE | Radiocarbon (from the same layer) | Mandibular left first incisor | 12,895,505 |
| SA-008 | 1384-936 calBCE | Radiocarbon (from the same layer) | Mandibular third right molar | 15,430,462 |
| SA-009 | 1900-1300 BCE (Middle Bronze Age) | Archaeological | Mandibular second left molar | 14,265,263 |
| SA-010 | 1900-1300 BCE (Middle Bronze Age) | Archaeological | Mandibular first right molar | 10,724,679 |
| SA-011 | 1900-1300 BCE (Middle Bronze Age) | Archaeological | Maxillary first left premolar | 14,632,189 |
| MAWB-3 | 2-sigma cal.BCE 170-CE 50* | Radiocarbon | Maxillary right lateral incisor | 2,730,685 |
| MAWB-5 | 2-sigma cal. 570-660 CE* | Radiocarbon | Mandibular right lateral incisor | 9,631,144 |
| MAWB-6 | 2-sigma cal. 713–885 CE** | Radiocarbon | Maxillary right central incisor | 32,402,210 |
| MAWB-10 | 830-1000 CE (Late-to-Terminal Classic) | Archaeological | Maxillary canine | 51,541,754 |
| MAWB-11 | 830-1000 CE (Late-to-Terminal Classic) | Archaeological | Maxillary canine | 11,999,444 |
| MAWB-12 | 830-1000 CE (Late-to-Terminal Classic) | Archaeological | Mandibular incisor | 8,652,497 |
| MAWB-15 | 830-1000 CE (Late-to-Terminal Classic) | Archaeological | Mandibular incisor | 35,754,125 |

Supplementary Table 8: Metagenome samples used in this study that were downnloaded from NCBI.

| **Sample Name** | **Dataset** | **Sample Type** | **Modern or Ancient** | **Article** | **Analysis Ready Reads** |
| --- | --- | --- | --- | --- | --- |
| Zape23 | RioZape | Coprolites | Ancient | Hagan et al. 2020 | 9047526 |
| Zape25 | RioZape | Coprolites | Ancient | Hagan et al. 2020 | 21126417 |
| Zape28 | RioZape | Coprolites | Ancient | Hagan et al. 2020 | 6942671 |
| Zape29 | RioZape | Coprolites | Ancient | Hagan et al. 2020 | 21134414 |
| Zape31 | RioZape | Coprolites | Ancient | Hagan et al. 2020 | 24219550 |
| Zape31 | RioZape | Coprolites | Ancient | Hagan et al. 2020 | 15571845 |
| Zape9 | RioZape | Coprolites | Ancient | Hagan et al. 2020 | 9473478 |
| ERR3003613 | Radcliffe | DentalCalculus | Ancient | Velsko et al. 2019 | 7432088 |
| ERR3003614 | Radcliffe | DentalCalculus | Ancient | Velsko et al. 2019 | 9224977 |
| ERR3003615 | Radcliffe | DentalCalculus | Ancient | Velsko et al. 2019 | 11888299 |
| ERR3003616 | Radcliffe | DentalCalculus | Ancient | Velsko et al. 2019 | 11147785 |
| ERR3003617 | Radcliffe | DentalCalculus | Ancient | Velsko et al. 2019 | 11375775 |
| ERR3003618 | Radcliffe | DentalCalculus | Ancient | Velsko et al. 2019 | 8856397 |
| ERR3003619 | Radcliffe | DentalCalculus | Ancient | Velsko et al. 2019 | 8443593 |
| ERR3003620 | Radcliffe | DentalCalculus | Ancient | Velsko et al. 2019 | 13444340 |
| ERR3003621 | Radcliffe | DentalCalculus | Ancient | Velsko et al. 2019 | 15568455 |
| ERR3003622 | Radcliffe | DentalCalculus | Ancient | Velsko et al. 2019 | 18881786 |
| ERR3003623 | Radcliffe | DentalCalculus | Ancient | Velsko et al. 2019 | 6976790 |
| ERR3003624 | Radcliffe | DentalCalculus | Ancient | Velsko et al. 2019 | 14396865 |
| ERR3003625 | Radcliffe | DentalCalculus | Ancient | Velsko et al. 2019 | 1636949 |
| ERR3003626 | Radcliffe | DentalCalculus | Ancient | Velsko et al. 2019 | 12959172 |
| ERR3003627 | Radcliffe | DentalCalculus | Ancient | Velsko et al. 2019 | 10735389 |
| ERR3003628 | Radcliffe | DentalCalculus | Ancient | Velsko et al. 2019 | 13671623 |
| ERR3003629 | Radcliffe | DentalCalculus | Ancient | Velsko et al. 2019 | 5619963 |
| ERR3003630 | Radcliffe | DentalCalculus | Ancient | Velsko et al. 2019 | 6892173 |
| ERR3003631 | Radcliffe | DentalCalculus | Ancient | Velsko et al. 2019 | 18613560 |
| ERR3003632 | Radcliffe | DentalCalculus | Ancient | Velsko et al. 2019 | 16636369 |
| ERR3003633 | Radcliffe | DentalCalculus | Ancient | Velsko et al. 2019 | 16505193 |
| ERR3003634 | Radcliffe | DentalCalculus | Ancient | Velsko et al. 2019 | 25529619 |
| ERR3003635 | Radcliffe | DentalCalculus | Ancient | Velsko et al. 2019 | 7250609 |
| ERR3003636 | Radcliffe | DentalCalculus | Ancient | Velsko et al. 2019 | 5037146 |
| ERR3003637 | Radcliffe | DentalCalculus | Ancient | Velsko et al. 2019 | 544226 |
| ERR3003638 | Radcliffe | DentalCalculus | Ancient | Velsko et al. 2019 | 13049172 |
| ERR3003639 | Radcliffe | DentalCalculus | Ancient | Velsko et al. 2019 | 20570653 |
| ERR3003640 | Radcliffe | DentalCalculus | Ancient | Velsko et al. 2019 | 5655198 |
| ERR3003641 | Radcliffe | DentalCalculus | Ancient | Velsko et al. 2019 | 21869403 |
| ERR3003642 | Radcliffe | DentalCalculus | Ancient | Velsko et al. 2019 | 29512892 |
| ERR3003643 | Radcliffe | DentalCalculus | Ancient | Velsko et al. 2019 | 11130624 |
| ERR3003644 | Radcliffe | DentalCalculus | Ancient | Velsko et al. 2019 | 8832898 |
| ERR3003645 | Radcliffe | DentalCalculus | Ancient | Velsko et al. 2019 | 27353094 |
| ERR3003646 | Radcliffe | DentalCalculus | Ancient | Velsko et al. 2019 | 7181939 |
| ERR3003647 | Radcliffe | DentalCalculus | Ancient | Velsko et al. 2019 | 22769227 |
| ERR3003648 | Radcliffe | DentalCalculus | Ancient | Velsko et al. 2019 | 14199232 |
| ERR3003649 | Radcliffe | DentalCalculus | Ancient | Velsko et al. 2019 | 9306260 |
| ERR3003650 | Radcliffe | DentalCalculus | Ancient | Velsko et al. 2019 | 4837871 |
| ERR3003651 | Radcliffe | DentalCalculus | Ancient | Velsko et al. 2019 | 5594629 |
| ERR3003652 | Radcliffe | DentalCalculus | Ancient | Velsko et al. 2019 | 8611932 |
| ERR3003653 | Radcliffe | DentalCalculus | Ancient | Velsko et al. 2019 | 10701129 |
| ERR3003654 | Radcliffe | DentalCalculus | Ancient | Velsko et al. 2019 | 4350720 |
| ERR3003655 | Radcliffe | DentalCalculus | Ancient | Velsko et al. 2019 | 9494263 |
| ERR3003656 | Radcliffe | DentalCalculus | Ancient | Velsko et al. 2019 | 29865341 |
| ERR3307045 | Spanish | DentalCalculus | Modern | Velsko et al. 2019 | 532661 |
| ERR3307046 | Spanish | DentalCalculus | Modern | Velsko et al. 2019 | 66116509 |
| ERR3307047 | Spanish | DentalCalculus | Modern | Velsko et al. 2019 | 50448700 |
| ERR3307048 | Spanish | DentalCalculus | Modern | Velsko et al. 2019 | 47951659 |
| ERR3307049 | Spanish | DentalCalculus | Modern | Velsko et al. 2019 | 49725788 |
| ERR3307050 | Spanish | DentalCalculus | Modern | Velsko et al. 2019 | 46646925 |
| ERR3307051 | Spanish | DentalCalculus | Modern | Velsko et al. 2019 | 44934750 |
| ERR3307052 | Spanish | DentalCalculus | Modern | Velsko et al. 2019 | 29167396 |
| ERR3307053 | Spanish | DentalCalculus | Modern | Velsko et al. 2019 | 49779231 |
| ERR3307054 | Spanish | DentalCalculus | Modern | Velsko et al. 2019 | 42609453 |
| HMP_700013715 | Human Microbiome Project | Feces | Modern | Methe et al. 2012 | 69559884 |
| HMP_700014562 | Human Microbiome Project | Feces | Modern | Methe et al. 2012 | 60328206 |
| HMP_700014724 | Human Microbiome Project | Feces | Modern | Methe et al. 2012 | 65768913 |
| HMP_700014837 | Human Microbiome Project | Feces | Modern | Methe et al. 2012 | 124239150 |
| HMP_700015113 | Human Microbiome Project | Feces | Modern | Methe et al. 2012 | 62790792 |
| HMP_700015181 | Human Microbiome Project | Feces | Modern | Methe et al. 2012 | 55171741 |
| HMP_700015250 | Human Microbiome Project | Feces | Modern | Methe et al. 2012 | 67801058 |
| HMP_700015415 | Human Microbiome Project | Feces | Modern | Methe et al. 2012 | 68904447 |
| HMP_700015857 | Human Microbiome Project | Feces | Modern | Methe et al. 2012 | 68373859 |
| HMP_700015922 | Human Microbiome Project | Feces | Modern | Methe et al. 2012 | 63686163 |
| HMP_700015981 | Human Microbiome Project | Feces | Modern | Methe et al. 2012 | 61297782 |
| HMP_700016142 | Human Microbiome Project | Feces | Modern | Methe et al. 2012 | 67429644 |
| HMP_700016456 | Human Microbiome Project | Feces | Modern | Methe et al. 2012 | 92884679 |
| HMP_700016542 | Human Microbiome Project | Feces | Modern | Methe et al. 2012 | 60661296 |
| HMP_700016610 | Human Microbiome Project | Feces | Modern | Methe et al. 2012 | 56801679 |
| HMP_700016765 | Human Microbiome Project | Feces | Modern | Methe et al. 2012 | 68981342 |
| HMP_700016960 | Human Microbiome Project | Feces | Modern | Methe et al. 2012 | 61064234 |
| HMP_700021306 | Human Microbiome Project | Feces | Modern | Methe et al. 2012 | 60102109 |
| HMP_700021824 | Human Microbiome Project | Feces | Modern | Methe et al. 2012 | 49572783 |
| HMP_700021876 | Human Microbiome Project | Feces | Modern | Methe et al. 2012 | 53859064 |
| HMP_700021902 | Human Microbiome Project | Feces | Modern | Methe et al. 2012 | 46993323 |
| HMP_700023113 | Human Microbiome Project | Feces | Modern | Methe et al. 2012 | 55727490 |
| HMP_700023267 | Human Microbiome Project | Feces | Modern | Methe et al. 2012 | 45389755 |
| HMP_700023337 | Human Microbiome Project | Feces | Modern | Methe et al. 2012 | 56015647 |
| HMP_700023578 | Human Microbiome Project | Feces | Modern | Methe et al. 2012 | 46214451 |
| HMP_700023634 | Human Microbiome Project | Feces | Modern | Methe et al. 2012 | 59541250 |
| HMP_700023720 | Human Microbiome Project | Feces | Modern | Methe et al. 2012 | 55382577 |
| HMP_700023845 | Human Microbiome Project | Feces | Modern | Methe et al. 2012 | 49526090 |
| HMP_700023872 | Human Microbiome Project | Feces | Modern | Methe et al. 2012 | 70233273 |
| HMP_700023919 | Human Microbiome Project | Feces | Modern | Methe et al. 2012 | 55612409 |
| HMP_700024024 | Human Microbiome Project | Feces | Modern | Methe et al. 2012 | 35149975 |
| HMP_700024233 | Human Microbiome Project | Feces | Modern | Methe et al. 2012 | 89685829 |
| HMP_700024318 | Human Microbiome Project | Feces | Modern | Methe et al. 2012 | 61627637 |
| HMP_700024437 | Human Microbiome Project | Feces | Modern | Methe et al. 2012 | 57012863 |
| HMP_700024449 | Human Microbiome Project | Feces | Modern | Methe et al. 2012 | 55469673 |
| HMP_700024509 | Human Microbiome Project | Feces | Modern | Methe et al. 2012 | 63665931 |
| HMP_700024615 | Human Microbiome Project | Feces | Modern | Methe et al. 2012 | 55080220 |
| HMP_700024673 | Human Microbiome Project | Feces | Modern | Methe et al. 2012 | 49800536 |
| HMP_700024711 | Human Microbiome Project | Feces | Modern | Methe et al. 2012 | 80926958 |
| HMP_700024752 | Human Microbiome Project | Feces | Modern | Methe et al. 2012 | 46935506 |
| HMP_700024866 | Human Microbiome Project | Feces | Modern | Methe et al. 2012 | 58765847 |
| HMP_700024930 | Human Microbiome Project | Feces | Modern | Methe et al. 2012 | 83543419 |
| HMP_700024998 | Human Microbiome Project | Feces | Modern | Methe et al. 2012 | 55518468 |
| HMP_700032222 | Human Microbiome Project | Feces | Modern | Methe et al. 2012 | 65325909 |
| HMP_700032244 | Human Microbiome Project | Feces | Modern | Methe et al. 2012 | 57218206 |
| HMP_700032338 | Human Microbiome Project | Feces | Modern | Methe et al. 2012 | 63596310 |
| HMP_700032944 | Human Microbiome Project | Feces | Modern | Methe et al. 2012 | 57182192 |
| HMP_700033153 | Human Microbiome Project | Feces | Modern | Methe et al. 2012 | 55130349 |
| HMP_700033435 | Human Microbiome Project | Feces | Modern | Methe et al. 2012 | 68709617 |
| HMP_700033502 | Human Microbiome Project | Feces | Modern | Methe et al. 2012 | 64224547 |
| HMP_700033665 | Human Microbiome Project | Feces | Modern | Methe et al. 2012 | 67213936 |
| Had1929408 | Hadza | Feces | Modern | Rampelli et al. 2015 | 31667557 |
| Had1929484 | Hadza | Feces | Modern | Rampelli et al. 2015 | 8023250 |
| Had1929485 | Hadza | Feces | Modern | Rampelli et al. 2015 | 4582373 |
| Had1929563 | Hadza | Feces | Modern | Rampelli et al. 2015 | 13965176 |
| Had1929574 | Hadza | Feces | Modern | Rampelli et al. 2015 | 10368513 |
| Had1930121 | Hadza | Feces | Modern | Rampelli et al. 2015 | 34860393 |
| Had1930122 | Hadza | Feces | Modern | Rampelli et al. 2015 | 15283833 |
| Had1930123 | Hadza | Feces | Modern | Rampelli et al. 2015 | 36600411 |
| Had1930128 | Hadza | Feces | Modern | Rampelli et al. 2015 | 14926939 |
| Had1930132 | Hadza | Feces | Modern | Rampelli et al. 2015 | 4278117 |
| Had1930133 | Hadza | Feces | Modern | Rampelli et al. 2015 | 4896636 |
| Had1930134 | Hadza | Feces | Modern | Rampelli et al. 2015 | 10999317 |
| Had1930136 | Hadza | Feces | Modern | Rampelli et al. 2015 | 13262019 |
| Had1930138 | Hadza | Feces | Modern | Rampelli et al. 2015 | 4698201 |
| Had1930140 | Hadza | Feces | Modern | Rampelli et al. 2015 | 7972145 |
| Had1930141 | Hadza | Feces | Modern | Rampelli et al. 2015 | 32205660 |
| Had1930142 | Hadza | Feces | Modern | Rampelli et al. 2015 | 5258350 |
| Had1930143 | Hadza | Feces | Modern | Rampelli et al. 2015 | 6562608 |
| Had1930144 | Hadza | Feces | Modern | Rampelli et al. 2015 | 4848670 |
| Had1930145 | Hadza | Feces | Modern | Rampelli et al. 2015 | 16560525 |
| Had1930149 | Hadza | Feces | Modern | Rampelli et al. 2015 | 4073672 |
| Had1930176 | Hadza | Feces | Modern | Rampelli et al. 2015 | 5041521 |
| Had1930177 | Hadza | Feces | Modern | Rampelli et al. 2015 | 5364707 |
| Had1930179 | Hadza | Feces | Modern | Rampelli et al. 2015 | 4013392 |
| Had1930187 | Hadza | Feces | Modern | Rampelli et al. 2015 | 3114848 |
| Had1930244 | Hadza | Feces | Modern | Rampelli et al. 2015 | 7743506 |
| bgi-N075A | China | Feces | Modern | Qin et al. 2012 | 36105871 |
| bgi-NLF002 | China | Feces | Modern | Qin et al. 2012 | 16058868 |
| bgi-NLF005 | China | Feces | Modern | Qin et al. 2012 | 19796667 |
| bgi-NLF006 | China | Feces | Modern | Qin et al. 2012 | 13659087 |
| bgi-NLF007 | China | Feces | Modern | Qin et al. 2012 | 22421737 |
| bgi-NLF009 | China | Feces | Modern | Qin et al. 2012 | 17280426 |
| bgi-NLF010 | China | Feces | Modern | Qin et al. 2012 | 16371986 |
| bgi-NLF011 | China | Feces | Modern | Qin et al. 2012 | 16745859 |
| bgi-NLF014 | China | Feces | Modern | Qin et al. 2012 | 17711182 |
| bgi-NLF015 | China | Feces | Modern | Qin et al. 2012 | 21249687 |
| bgi-NLM006 | China | Feces | Modern | Qin et al. 2012 | 25751198 |
| bgi-NLM010 | China | Feces | Modern | Qin et al. 2012 | 24699774 |
| bgi-NLM015 | China | Feces | Modern | Qin et al. 2012 | 14397119 |
| bgi-NLM016 | China | Feces | Modern | Qin et al. 2012 | 13553365 |
| bgi-NLM022 | China | Feces | Modern | Qin et al. 2012 | 21914212 |
| bgi-NLM023 | China | Feces | Modern | Qin et al. 2012 | 27269972 |
| bgi-NLM027 | China | Feces | Modern | Qin et al. 2012 | 23760705 |
| bgi-NLM028 | China | Feces | Modern | Qin et al. 2012 | 23235883 |
| bgi-NLM029 | China | Feces | Modern | Qin et al. 2012 | 23089593 |
| bgi-NLM031 | China | Feces | Modern | Qin et al. 2012 | 23091231 |
| bgi-NOF002 | China | Feces | Modern | Qin et al. 2012 | 20696915 |
| bgi-NOF005 | China | Feces | Modern | Qin et al. 2012 | 22765953 |
| bgi-NOF008 | China | Feces | Modern | Qin et al. 2012 | 24152020 |
| bgi-NOF009 | China | Feces | Modern | Qin et al. 2012 | 21438264 |
| bgi-NOF012 | China | Feces | Modern | Qin et al. 2012 | 19714306 |
| bgi-NOF013 | China | Feces | Modern | Qin et al. 2012 | 21137523 |
| bgi-NOF014 | China | Feces | Modern | Qin et al. 2012 | 17546823 |
| bgi-NOM001 | China | Feces | Modern | Qin et al. 2012 | 14058605 |
| bgi-NOM004 | China | Feces | Modern | Qin et al. 2012 | 20126357 |
| bgi-NOM007 | China | Feces | Modern | Qin et al. 2012 | 14905722 |
| bgi-NOM009 | China | Feces | Modern | Qin et al. 2012 | 22994893 |
| bgi-NOM017 | China | Feces | Modern | Qin et al. 2012 | 17256657 |
| bgi-NOM018 | China | Feces | Modern | Qin et al. 2012 | 21390737 |
| bgi-NOM019 | China | Feces | Modern | Qin et al. 2012 | 15779040 |
| bgi-NOM020 | China | Feces | Modern | Qin et al. 2012 | 20163678 |
| bgi-NOM023 | China | Feces | Modern | Qin et al. 2012 | 17845086 |
| bgi-NOM027 | China | Feces | Modern | Qin et al. 2012 | 17335223 |
| bgi-NOM028 | China | Feces | Modern | Qin et al. 2012 | 19197467 |
| SM01 | Matses | Feces | Modern | Obregon-Tito et al. 2015 | 36243326 |
| SM02 | Matses | Feces | Modern | Obregon-Tito et al. 2015 | 46085816 |
| SM03 | Matses | Feces | Modern | Obregon-Tito et al. 2015 | 22843103 |
| SM05 | Matses | Feces | Modern | Obregon-Tito et al. 2015 | 23906323 |
| SM10 | Matses | Feces | Modern | Obregon-Tito et al. 2015 | 27723664 |
| SM11 | Matses | Feces | Modern | Obregon-Tito et al. 2015 | 33348829 |
| SM18 | Matses | Feces | Modern | Obregon-Tito et al. 2015 | 32026148 |
| SM20 | Matses | Feces | Modern | Obregon-Tito et al. 2015 | 30099313 |
| SM23 | Matses | Feces | Modern | Obregon-Tito et al. 2015 | 30146410 |
| SM24 | Matses | Feces | Modern | Obregon-Tito et al. 2015 | 32991287 |
| SM25 | Matses | Feces | Modern | Obregon-Tito et al. 2015 | 31026199 |
| SM28 | Matses | Feces | Modern | Obregon-Tito et al. 2015 | 27389507 |
| SM29 | Matses | Feces | Modern | Obregon-Tito et al. 2015 | 30151973 |
| SM30 | Matses | Feces | Modern | Obregon-Tito et al. 2015 | 27805060 |
| SM31 | Matses | Feces | Modern | Obregon-Tito et al. 2015 | 30133715 |
| SM32 | Matses | Feces | Modern | Obregon-Tito et al. 2015 | 35061331 |
| SM33 | Matses | Feces | Modern | Obregon-Tito et al. 2015 | 28563888 |
| SM34 | Matses | Feces | Modern | Obregon-Tito et al. 2015 | 26961514 |
| SM37 | Matses | Feces | Modern | Obregon-Tito et al. 2015 | 27433220 |
| SM39 | Matses | Feces | Modern | Obregon-Tito et al. 2015 | 33510372 |
| SM40 | Matses | Feces | Modern | Obregon-Tito et al. 2015 | 33098422 |
| SM41 | Matses | Feces | Modern | Obregon-Tito et al. 2015 | 30877682 |
| SM42 | Matses | Feces | Modern | Obregon-Tito et al. 2015 | 28698309 |
| SM43 | Matses | Feces | Modern | Obregon-Tito et al. 2015 | 31343591 |
| SM44 | Matses | Feces | Modern | Obregon-Tito et al. 2015 | 31275968 |

**V. Supplementary References**

[1] Webster, D. 2002 *The fall of the ancient Mayasolving the mystery of the Maya collapse*.

[2] Booher, A. M. 2016 Assessing the form and function of the Sacbeob and associated structures at Chan Chich, Belize.

[3] Houk, B. A. The Chan Chich Archaeological Project: 1996 to 2019 Project Lists. In *THE 2019 SEASONS OF THE BELIZE ESTATES ARCHAEOLOGICAL SURVEY TEAM* (ed. B. A. Houk), pp. 169-208. Papers of the Chan Chich Archaeological Project, Number 14, Department of Sociology, Anthropology, and Social Work, Texas Tech University, Lubbock.

[4] Kosakowsky, L. J. & Robin, C. 2012 Ceramics and chronology at Chan. *Chan: an ancient Maya farming community*, 42-70.

[5] Novotny, A. & Robin, C. 2012 The Chan community: A bioarchaeological perspective. *Chan: An ancient Maya farming community*, 231-252.

[6] Fernandes, D. M., Mittnik, A., Olalde, I., Lazaridis, I., Cheronet, O., Rohland, N., Mallick, S., Bernardos, R., Broomandkhoshbacht, N., Carlsson, J., et al. 2020 The spread of steppe and Iranian-related ancestry in the islands of the western Mediterranean. *Nat Ecol Evol* **4**, 334-345. (DOI:10.1038/s41559-020-1102-0).

[7] Ucchesu, M., Peña-Chocarro, L., Sabato, D. & Tanda, G. 2015 Bronze Age subsistence in Sardinia, Italy: cultivated plants and wild resources. *Vegetation history and archaeobotany* **24**, 343-355.

[8] Portas, L., Bagella, S., Farina, V., Carcupino, M., Cacchioli, A., Gazza, F. & Zedda, M. 2015 Study of animal remains dug out during the excavations of a Nuragic village in Sardinia. *Journal of Biological Research-Bollettino della Società Italiana di Biologia Sperimentale*.

[9] Lai, L., Tykot, R. H., Usai, E., Beckett, J. F., Floris, R., Fonzo, O., Goddard, E., Hollander, D., Manunza, M. R. & Usai, A. 2013 Diet in the Sardinian Bronze Age: models, collagen isotopic data, issues and perspectives. *Préhistoires Méditerranéennes*.

[10] Sarigu, M., Floris, G., Floris, R. & Pusceddu, V. 2016 The Osteological Collection of the University of Cagliari: From Early Neolithic to Modern Age. *HOMO* **67**, 216-225.

[11] Cooper, A. & Poinar, H. N. 2000 Ancient DNA: do it right or not at all. *Science* **289**, 1139-1139.

[12] Ozga, A. T., Nieves‐Colón, M. A., Honap, T. P., Sankaranarayanan, K., Hofman, C. A., Milner, G. R., Lewis Jr, C. M., Stone, A. C. & Warinner, C. 2016 Successful enrichment and recovery of whole mitochondrial genomes from ancient human dental calculus. *American journal of physical anthropology* **160**, 220-228.

[13] Rohland, N., Harney, E., Mallick, S., Nordenfelt, S. & Reich, D. 2015 Partial uracil–DNA–glycosylase treatment for screening of ancient DNA. *Philosophical Transactions of the Royal Society B: Biological Sciences* **370**, 20130624.

[14] Schubert, M., Lindgreen, S. & Orlando, L. 2016 AdapterRemoval v2: rapid adapter trimming, identification, and read merging. *BMC research notes* **9**, 88.

[15] DeSantis, T. Z., Hugenholtz, P., Larsen, N., Rojas, M., Brodie, E. L., Keller, K., Huber, T., Dalevi, D., Hu, P. & Andersen, G. L. 2006 Greengenes, a chimera-checked 16S rRNA gene database and workbench compatible with ARB. *Appl. Environ. Microbiol.* **72**, 5069-5072.

[16] Langmead, B. & Salzberg, S. L. 2012 Fast gapped-read alignment with Bowtie 2. *Nature methods* **9**, 357.

[17] Li, H., Handsaker, B., Wysoker, A., Fennell, T., Ruan, J., Homer, N., Marth, G., Abecasis, G. & Durbin, R. 2009 The sequence alignment/map format and SAMtools. *Bioinformatics* **25**, 2078-2079.

[18] Caporaso, J. G., Kuczynski, J., Stombaugh, J., Bittinger, K., Bushman, F. D., Costello, E. K., Fierer, N., Pena, A. G., Goodrich, J. K. & Gordon, J. I. 2010 QIIME allows analysis of high-throughput community sequencing data. *Nature methods* **7**, 335.

[19] Knights, D., Kuczynski, J., Charlson, E. S., Zaneveld, J., Mozer, M. C., Collman, R. G., Bushman, F. D., Knight, R. & Kelley, S. T. 2011 Bayesian community-wide culture-independent microbial source tracking. *Nature methods* **8**, 761.

[20] Jónsson, H., Ginolhac, A., Schubert, M., Johnson, P. L. & Orlando, L. 2013 mapDamage2. 0: fast approximate Bayesian estimates of ancient DNA damage parameters. *Bioinformatics* **29**, 1682-1684.

[21] Li, H. & Durbin, R. 2009 Fast and accurate short read alignment with Burrows–Wheeler transform. *bioinformatics* **25**, 1754-1760.

[22] Schubert, M., Ginolhac, A., Lindgreen, S., Thompson, J. F., Al-Rasheid, K. A., Willerslev, E., Krogh, A. & Orlando, L. 2012 Improving ancient DNA read mapping against modern reference genomes. *BMC genomics* **13**, 178.

[23] Peltzer, A., Jäger, G., Herbig, A., Seitz, A., Kniep, C., Krause, J. & Nieselt, K. 2016 EAGER: efficient ancient genome reconstruction. *Genome biology* **17**, 60.

[24] Truong, D. T., Franzosa, E. A., Tickle, T. L., Scholz, M., Weingart, G., Pasolli, E., Tett, A., Huttenhower, C. & Segata, N. 2015 MetaPhlAn2 for enhanced metagenomic taxonomic profiling. *Nature methods* **12**, 902-903.

[25] Franzosa, E. A., McIver, L. J., Rahnavard, G., Thompson, L. R., Schirmer, M., Weingart, G., Lipson, K. S., Knight, R., Caporaso, J. G. & Segata, N. 2018 Species-level functional profiling of metagenomes and metatranscriptomes. *Nature methods* **15**, 962-968.

[26] Suzek, B. E., Huang, H., McGarvey, P., Mazumder, R. & Wu, C. H. 2007 UniRef: comprehensive and non-redundant UniProt reference clusters. *Bioinformatics* **23**, 1282-1288.

[27] Team, R. C. 2013 R: A language and environment for statistical computing.

[28] Layeghifard, M., Hwang, D. M. & Guttman, D. S. 2018 Constructing and Analyzing Microbiome Networks in R. In *Microbiome Analysis* (pp. 243-266, Springer.

[29] Kurtz, Z., Mueller, C., Miraldi, E. & Bonneau, R. 2017 SpiecEasi: Sparse Inverse Covariance for Ecological Statistical Inference. *R package version* **1**.

[30] Csardi, G. & Nepusz, T. 2006 The igraph software package for complex network research. *InterJournal, complex systems* **1695**, 1-9.

[31] Yoon, S.-H., Ha, S.-M., Kwon, S., Lim, J., Kim, Y., Seo, H. & Chun, J. 2017 Introducing EzBioCloud: a taxonomically united database of 16S rRNA gene sequences and whole-genome assemblies. *International journal of systematic and evolutionary microbiology* **67**, 1613.

[32] Katoh, K. & Standley, D. M. 2013 MAFFT multiple sequence alignment software version 7: improvements in performance and usability. *Molecular biology and evolution* **30**, 772-780.

[33] Price, M. N., Dehal, P. S. & Arkin, A. P. 2010 FastTree 2–approximately maximum-likelihood trees for large alignments. *PloS one* **5**.

[34] Oksanen, J., Blanchet, F. G., Kindt, R., Legendre, P., O’hara, R., Simpson, G. L., Solymos, P., Stevens, M. H. H. & Wagner, H. 2010 Vegan: community ecology package. R package version 1.17-4. *URL* [*http://CRAN*](http://CRAN)*. R-project. org/package= vegan*.

[35] Kembel, S. W., Cowan, P. D., Helmus, M. R., Cornwell, W. K., Morlon, H., Ackerly, D. D., Blomberg, S. P. & Webb, C. O. 2010 Picante: R tools for integrating phylogenies and ecology. *Bioinformatics* **26**, 1463-1464.

[36] Wickham, H. 2016 *ggplot2: elegant graphics for data analysis*, Springer.

[37] Benjamini, Y. & Hochberg, Y. 1995 Controlling the false discovery rate: a practical and powerful approach to multiple testing. *Journal of the Royal statistical society: series B (Methodological)* **57**, 289-300.

[38] Faust, K., Bauchinger, F., Laroche, B., De Buyl, S., Lahti, L., Washburne, A. D., Gonze, D. & Widder, S. 2018 Signatures of ecological processes in microbial community time series. *Microbiome* **6**, 1-13.

[39] McNally, L. & Brown, S. P. 2016 Microbiome: Ecology of stable gut communities. *Nature microbiology* **1**, 1-2.

[40] Stein, R. R., Bucci, V., Toussaint, N. C., Buffie, C. G., Rätsch, G., Pamer, E. G., Sander, C. & Xavier, J. B. 2013 Ecological modeling from time-series inference: insight into dynamics and stability of intestinal microbiota. *PLoS computational biology* **9**.

[41] Layeghifard, M., Hwang, D. M. & Guttman, D. S. 2017 Disentangling interactions in the microbiome: a network perspective. *Trends in microbiology* **25**, 217-228.

[42] Tian, L., Bashan, A., Shi, D.-N. & Liu, Y.-Y. 2017 Articulation points in complex networks. *Nature communications* **8**, 1-9.

[43] Gloor, G. B., Macklaim, J. M., Pawlowsky-Glahn, V. & Egozcue, J. J. 2017 Microbiome datasets are compositional: and this is not optional. *Frontiers in microbiology* **8**, 2224.

[44] Tsilimigras, M. C. & Fodor, A. A. 2016 Compositional data analysis of the microbiome: fundamentals, tools, and challenges. *Annals of epidemiology* **26**, 330-335.

[45] Fang, H., Huang, C., Zhao, H. & Deng, M. 2017 gCoda: conditional dependence network inference for compositional data. *Journal of Computational Biology* **24**, 699-708.

[46] Friedman, J. & Alm, E. J. 2012 Inferring correlation networks from genomic survey data. *PLoS computational biology* **8**.

[47] Kurtz, Z. D., Müller, C. L., Miraldi, E. R., Littman, D. R., Blaser, M. J. & Bonneau, R. A. 2015 Sparse and compositionally robust inference of microbial ecological networks. *PLoS computational biology* **11**.

[48] Faust, K. & Raes, J. 2012 Microbial interactions: from networks to models. *Nature Reviews Microbiology* **10**, 538-550.

[49] Barber, M. J. 2007 Modularity and community detection in bipartite networks. *Physical Review E* **76**, 066102.

[50] Newman, M. E. 2006 Modularity and community structure in networks. *Proceedings of the national academy of sciences* **103**, 8577-8582.

[51] Page, L., Brin, S., Motwani, R. & Winograd, T. 1997 PageRank: Bringing order to the web. (Stanford Digital Libraries Working Paper.

[52] Xing, W. & Ghorbani, A. 2004 Weighted pagerank algorithm. In *Proceedings. Second Annual Conference on Communication Networks and Services Research, 2004.* (pp. 305-314, IEEE.

[53] Brandes, U., Borgatti, S. P. & Freeman, L. C. 2016 Maintaining the duality of closeness and betweenness centrality. *Social Networks* **44**, 153-159.

[54] Banerjee, S., Schlaeppi, K. & van der Heijden, M. G. 2018 Keystone taxa as drivers of microbiome structure and functioning. *Nature Reviews Microbiology* **16**, 567-576.

[55] Cappellini, E., Prohaska, A., Racimo, F., Welker, F., Pedersen, M. W., Allentoft, M. E., de Barros Damgaard, P., Gutenbrunner, P., Dunne, J., Hammann, S., et al. 2018 Ancient Biomolecules and Evolutionary Inference. *Annu Rev Biochem* **87**, 1029-1060. (DOI:10.1146/annurev-biochem-062917-012002).

[56] Velsko, I. M., Yates, J. A. F., Aron, F., Hagan, R. W., Frantz, L. A., Loe, L., Martinez, J. B. R., Chaves, E., Gosden, C. & Larson, G. 2019 Microbial differences between dental plaque and historic dental calculus are related to oral biofilm maturation stage. *Microbiome* **7**, 102.
